# miRNA-132/212 Deficiency Disrupts Selective Corticosterone Modulation of Dorsal vs. Ventral Hippocampal Metaplasticity

**DOI:** 10.3390/ijms24119565

**Published:** 2023-05-31

**Authors:** Shima Kouhnavardi, Maureen Cabatic, M. Carmen Mañas-Padilla, Marife-Astrid Malabanan, Tarik Smani, Ana Cicvaric, Edison Alejandro Muñoz Aranzalez, Xaver Koenig, Ernst Urban, Gert Lubec, Estela Castilla-Ortega, Francisco J. Monje

**Affiliations:** 1 Center for Physiology and Pharmacology, Department of Neurophysiology and Neuropharmacology, Medical University of Vienna, 1090 Vienna, Austria; 2 Instituto de Investigación Biomédica de Málaga-IBIMA, 29590 Málaga, Spain; 3 Department of Medical Physiology and Biophysics, University of Seville, 41013 Seville, Spain; 4 Dominick P. Purpura Department of Neuroscience, Albert Einstein College of Medicine, New York, NY 10461, USA; 5 Department for Pharmaceutical Sciences, Josef-Holaubek-Platz 2, 2D 303, 1090 Vienna, Austria; 6 Programme for Proteomics, Paracelsus Medical University, 5020 Salzburg, Austria

**Keywords:** microRNA, miR-132/212, synaptic plasticity, dorsal hippocampus, ventral hippocampus, corticosterone, emotional behavior, anxiety-like behavior

## Abstract

Cortisol is a potent human steroid hormone that plays key roles in the central nervous system, influencing processes such as brain neuronal synaptic plasticity and regulating the expression of emotional and behavioral responses. The relevance of cortisol stands out in the disease, as its dysregulation is associated with debilitating conditions such as Alzheimer’s Disease, chronic stress, anxiety and depression. Among other brain regions, cortisol importantly influences the function of the hippocampus, a structure central for memory and emotional information processing. The mechanisms fine-tuning the different synaptic responses of the hippocampus to steroid hormone signaling remain, however, poorly understood. Using ex vivo electrophysiology and wild type (WT) and miR-132/miR-212 microRNAs knockout (miRNA-132/212^−/−^) mice, we examined the effects of corticosterone (the rodent’s equivalent to cortisol in humans) on the synaptic properties of the dorsal and ventral hippocampus. In WT mice, corticosterone predominantly inhibited metaplasticity in the dorsal WT hippocampi, whereas it significantly dysregulated both synaptic transmission and metaplasticity at dorsal and ventral regions of miR–132/212^−/−^ hippocampi. Western blotting further revealed significantly augmented levels of endogenous CREB and a significant CREB reduction in response to corticosterone only in miR–132/212^−/−^ hippocampi. Sirt1 levels were also endogenously enhanced in the miR–132/212^−/−^ hippocampi but unaltered by corticosterone, whereas the levels of phospo-MSK1 were only reduced by corticosterone in WT, not in miR–132/212^−/−^ hippocampi. In behavioral studies using the elevated plus maze, miRNA-132/212^−/−^ mice further showed reduced anxiety-like behavior. These observations propose miRNA-132/212 as potential region-selective regulators of the effects of steroid hormones on hippocampal functions, thus likely fine-tuning hippocampus-dependent memory and emotional processing.

## 1. Introduction

The steroid hormone cortisol (also known as hydrocortisone) is a highly potent human hormone produced by the adrenal glands and whose secretion into the blood stream is triggered by the corticotropin-releasing hormone originating from the hypothalamus in the brain. Cortisol is central for the regulation of many biological functions, including the modulation of the body responses to stress, the reduction of inflammatory processes, the regulation of blood pressure and metabolic functions, and the influences on the immune system and circadian sleep-wake cycles [1,2]. Cortisol is also a powerful regulator of brain function, influencing learning, memory and emotional behaviors in both humans and animals [2,3,4,5]. Moreover, dysregulated cortisol levels have been identified during the onset and progression of a variety of conditions associated with memory decline and emotional dysregulation, including post-traumatic stress disorder, depression, stress and anxiety [6,7,8,9,10,11,12,13,14,15]. Several studies have also shown that altered levels of corticosterone (the molecular counterpart of cortisol in mice and rats) result in profound changes in neuronal synaptic plasticity as well as altered memory-related emotional behaviors [16,17,18,19,20,21,22]. The molecular mechanisms by which cortisol can distinctly affect the different regions of the brain to influence memory and emotion-related behaviors remain, however, poorly understood.

The hippocampus, a brain structure critical for learning and memory processing [23,24,25,26], is involved in the regulation of emotional memories [27,28,29] and is importantly associated with the modulation of the hypothalamic–pituitary–adrenal axis function in health and disease [30,31,32,33]. Interestingly, exposure to early-life stress has been shown to correlate with a reduction in the anatomical features of hippocampal structures as well as alterations in cortisol levels [30,33]. Moreover, while the hippocampus is the brain region with perhaps the highest concentration of receptor target sites for adrenocortical steroids [29], and whereas several studies have shown that corticosterone can directly influence hippocampal synaptic potentiation [34,35], little continues to be known about how cortisol differentially affects the different regions of the hippocampus to influence memory-related emotional behaviors [28]. Here, we hypothesized that microRNAs (miRNAs) are key molecular elements participating in the modulation of the region-specific effects of glucocorticoid hormones on hippocampal synaptic transmission and metaplasticity (the newly generated synaptic changes that happen after synaptic plasticity has occurred [36,37,38]).

miRNAs, are non-encoding short transcript RNAs that participate in the posttranscriptional modulation of gene expression [39,40]; that serve in a variety of pivotal functions of the central nervous systems, including the modulation of synaptic activity [41,42,43,44,45,46]; and which are importantly involved in the brain neuropathology [47,48,49,50,51]. Elevated levels of some miRNAs (e.g., miR-455-3p), have been found in serum samples from Alzheimer’s disease patients relative to those obtained from healthy controls, thus suggesting the possibility that miR-455-3p and other miRNAs [49] could even be used as biomarkers in the contexts of very severe neuropathologies, as is the case of Alzheimer’s disease [48]. In line with this, specific studies of the levels of the miRNA miR-132 have been conducted using quantitative real time PCR in postmortem brain tissue samples obtained from deceased patients that had being diagnosed with Alzheimer’s disease and with mild cognitive-decline. These studies showed significantly enhanced levels of miR-132 in the samples from both Alzheimer’s disease and cognitive-decline groups as compared with the data obtained from their normal (control) samples, suggesting that miR-132 might be a critical player in the pathogenesis of Alzheimer’s disease [47]. Interestingly, other authors have described not elevated but rather markedly reduced levels of miR-132 at middle and advanced stages of Alzheimer’s disease [50], and that the reduction in this specific miRNA aggravates amyloid and TAU pathological features [52]. For many years, miR-132 has been jointly examined together with miR-212 in a variety of different multidisciplinary studies (see for example [53,54,55,56,57,58]), as both these molecules are described to belong to the same highly conserved cluster family of miRNAs derived from a shared phylogenetic ancestor; further having their gene chromosomic organization arrayed as a tandem [59]. Similarly, in experiments using genetically modified mice, the combined genetic deletion of the genes encoding for miR-132 and miR-212 has been shown to result in an enhancement in the levels of amyloid beta peptides as well as in augmented amyloid-related plaque formation [51]. All these observations had clearly identified the microRNA212/132 cluster family as highly interesting candidates in studies about the brain neuronal function in health and disease.

Several groups, including ours, have previously described some of the functional effects of the double-deletion of the genes encoding for the miRNAs 132 and 212 in the regulation of plasticity-related functions in the mouse hippocampus [43,57,60,61]. However, although both steroid-hormones, including cortisol and corticosterone, as well as the miRNAs 132 and 212, have been independently implicated in the regulation of synaptic plasticity and emotion-related functions ([62,63]), the potential functional crosslink between steroid hormones; brain region-specific regulation of synaptic plasticity; and the miRNAs 132 and 212, remained -to our knowledge- unexplored. Therefore, the main objective of this work was to use a previously described double miR-132 and miR-212 miRNAs knockout mouse line [56] (here referred as miRNA-132/212^−/−^, or KO), in order to electrophysiologically study the synaptic responses to corticosterone stimulation in hippocampal slices, and to examine whether these responses are functionally homogeneous or not when measurements at the dorsal and ventral hippocampi are compared. We have obtained experimental data suggesting a potential role for the miR-132/12 in hippocampal neuroendocrine signaling and emotion-related behaviors.

## 2. Results

### 2.1. Comparable Basal Synaptic Transmission in Dorsal and Ventral Hippocampi of WT and miR–132/212^−/−^ Mice

In order to examine the physiological relevance of the microRNAs 132 and 212 in mediating the hippocampal synaptic responses to corticosterone stimulation, we explored the effect of 1 µM corticosterone on basal synaptic transmission and memory-related synaptic plasticity in the dorsal and ventral hippocampus of wild type and miR–132/212^−/−^ mice. To this aim, we conducted recordings of extracellular field potentials ex vivo in acutely-dissociated hippocampal slices following standardized electrophysiological protocols previously described by our group and others [43,60,61,64,65,66,67,68,69,70].

**Figure 1:**
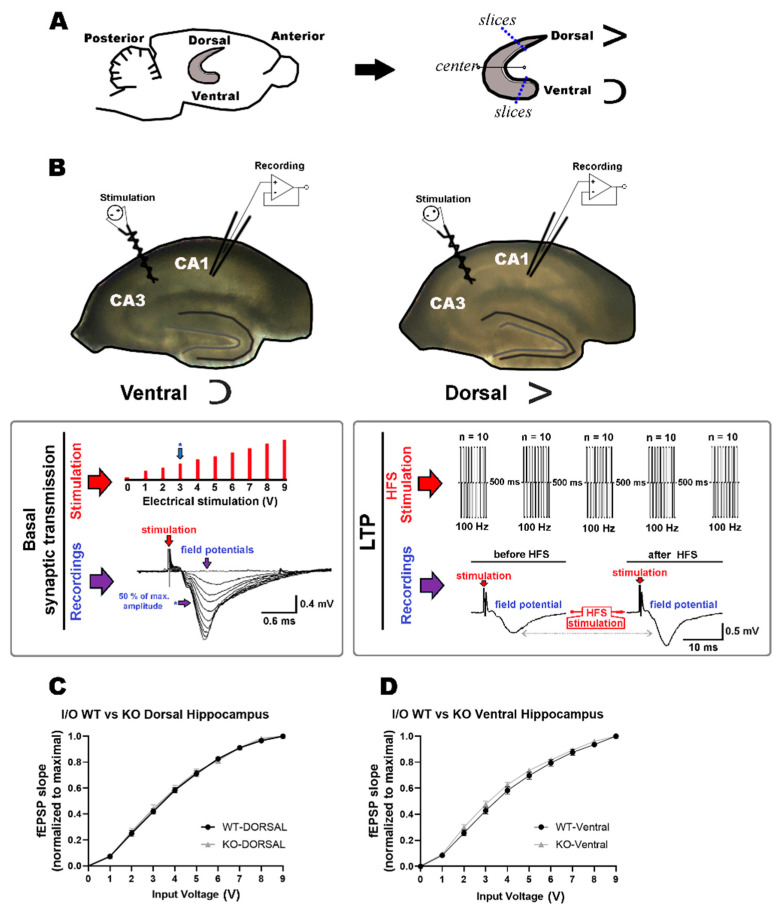
Description of the electrophysiological approach and study of basal synaptic transmission. (**A**) The cartoon on the left represents a sagittal section of the mouse brain with the relative localization of one of the two hippocampi and Anterior–Posterior and Dorsal–Ventral references indicated. To the right, the black arrow points towards the extracted hippocampus, which was divided into two parts using its anatomical middle as the nominal center. Slices were subsequently obtained from the ending dorsal (represented by a left-90°-rotated “V” shape) and ventral (represented by a horizontally flipped “C” shape) regions that comprise circa 30–40% (blue dotted lines) of the portion located longitudinally from the center towards each respective end. (**B**) Two representative microphotographs of hippocampal slices from the ventral (**left**) and dorsal (**right**) regions were used to hereby illustrate the synaptic regions examined electrophysiologically and the positioning of the recording and stimulating electrodes. The tossed “V”- and “C”-like black lines partially surrounding the dentate gyrus (gray line inside the slices) illustrate the morphology typically observed ventrally and dorsally under the microscope. The CA3 and CA1 regions are indicated. In the large, boxed inset below (**left**): Schematic representation of the protocol used to generate input/output (I/O) curves used to assess basal synaptic transmission. The large, red-filled arrow at the top left points towards the delivered electrical stimulation pulses, which consisted of 10 discrete 200 μs voltage steps (in red) from 0–9 V delivered with 15 s intervals. Below, the large purple-filled arrow on the left points towards representative traces of the 10 elicited field potential recordings. The small blue arrow preceded by an asterisk indicates a field recording with an amplitude of approximately 50% of the maximal achievable amplitude, which, in this diagram, and as an illustration, would have been generated by the step of voltage stimulation of 3 V, as indicated in the upper panel by a small blue arrow preceded by an asterisk. In the large, boxed inset below (**right**): Schematic representation of the high-frequency stimulation (HFS) protocol used to generate long-term potentiation (LTP). In the top panel, the large, red-filled arrow on the left points towards the 5 delivered bursts of electrical stimulation pulses, each burst consisting of 10 biphasic voltage steps (100 μs/phase, 100 Hz, with 500 ms intervals) given at voltage intensities eliciting about 50% of the maximal inducible amplitude. In the lower panel, the large, purple-filled arrow on the left points towards representative traces illustrating elicited field potential recordings during the baseline recordings (before HFS) and after having delivered the LTP-inducing protocol (after HFS). During experiments, the “before” and “after” field recordings are obtained upon delivering one biphasic pule (100 μs/phase) with inter-stimulus intervals of 30 s, using the same voltage stimulation that elicited about 50% of the maximal inducible amplitude. (**C**,**D**) The line charts represent the data from the changes in the field slopes versus the different values of voltage delivered (normalized to maximal slope) for slices from the dorsal and ventral regions, respectively, obtained from WT and miRNA-132/212^−/−^ mice (*n* = 21 animals per group). No statistically significant differences were observed between the different groups (details in the main text). Data are shown as mean ± SEM.

As illustrated in Figure 1A, for these experiments, we extracted the hippocampus and split it into two main pieces lengthwise the septo-temporal axis, relative to the morphological transverse middle, and then slices for electrophysiological recordings were obtained from about 30–40% of the section comprising the defined center towards either the dorsal or the ventral ending regions (longitudinally). These areas are known to exhibit classical LTP responses as induced by electrical stimulation [71,72]. Subsequently, field potential recordings were conducted at the CA1 region upon delivering electrical stimulation at the Schaffer’s collaterals from the CA3 region for both dorsal and ventral regions (Figure 1B). For plasticity experiments, recordings were conducted in the presence and absence of corticosterone in slices from both WT and miR–132/212^−/−^ mice. Corticosterone was applied in the bath for the time specified in the figures and then removed using gravity-based-perfusion and peristaltic pump-driven solution exchange. Before examining the effect of 1 µM corticosterone on the properties of synaptic plasticity, we verified the functional integrity of the synaptic circuits by measuring input/output (I/O) field responses (see Materials and Methods) in untreated dorsal and ventral hippocampi of WT and miR–132/212^−/−^ mice. As shown in Figure 1CD, no statistically significant differences were found in basal synaptic transmission when comparing the data obtained in slices derived from WT and miR–132/212^−/−^ mice. Two-way RM-ANOVA with Bonferroni´s and Geisser–Greenhouse´s corrections, and alpha set to 0.05 (*n* = 21 animals per group), showed no significance for the effects of the 1 µM corticosterone treatment for WT vs. miR–132/212^−/−^ at dorsal (*p* = 0.7450; F _(1, 40)_ = 0.1073) or ventral (*p* = 0.1748; F _(1, 40)_ = 1.908) hippocampi.

### 2.2. miRNA-132/212 Gene-Depletion Differentially Affects Short-Term Plasticity in Ventral and Dorsal Hippocampus

In order to further characterize the functional activity at the dorsal and ventral hippocampi of WT and miR–132/212^−/−^ mice, we sought to investigate the properties of presynaptic-dependent short-term plasticity. To this aim, we implemented electrophysiological protocols of Paired-Pulse-induced Facilitation (PPF) in brain slices following methods previously described by our group and others [60,61,73,74] (see also Materials and Methods). Through PPF protocols, it is possible to experimentally evoke a form of short-term plasticity that has been functionally associated with the modulation of exocytosis [75] and which is further proposed to influence the properties of long-term forms of synaptic plasticity [76].

**Figure 2:**
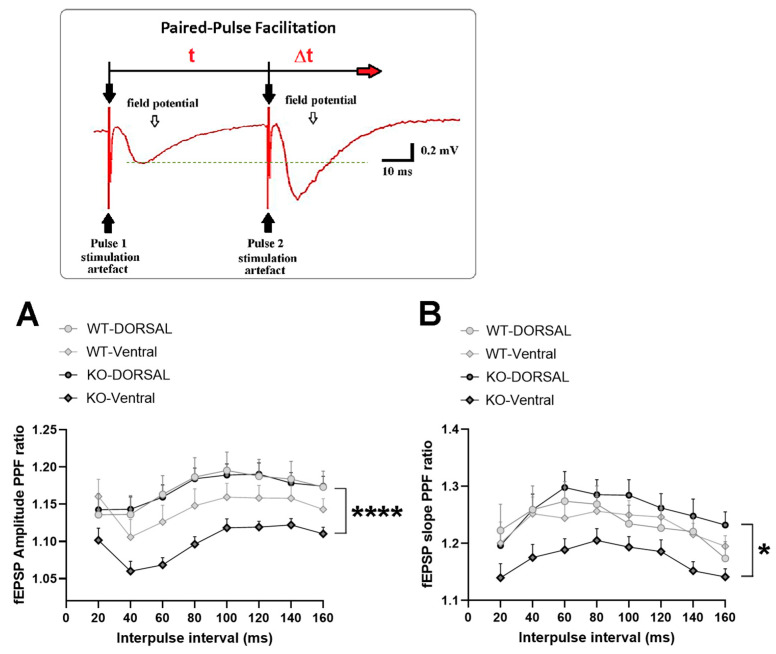
miRNA-132/212 gene-deletion influences presynaptic-dependent short-term facilitation in the mouse hippocampus. The cartoon in the upper inset illustrates the paired-pulse facilitation protocol used to induce short-term facilitation. Two consecutive pulses of electrical stimulation (indicated by black filled arrows) were delivered with an initial 20 ms interpulse interval (t), followed by consecutive increments in 20 ms (Δt) of duration until reaching 160 ms. The red-filled arrow pointing towards the right indicates the advance in time. Ratios for the values of raw amplitude or initial decay slope of the field potential responses (EPSP_2_/EPSP_1_) were used to quantify the power of paired-pulse-induced facilitation. No major differences are detected in the PPF amplitude (**A**) and field-slope ratios (**B**) when recordings from the dorsal and ventral hippocampi are examined in response to the different interpulse time intervals in slices from WT animals. Conversely, a marked difference is observed in the properties of the PPF amplitude, and field-slope ratios are examined in slices derived from miR–132/212^−/−^ mice. *p* < 0.05 was considered significant. * *p* < 0.05, **** *p* < 0.0001. Data are shown as mean ± SEM. A total of 21–22 animals per group were examined. Statistical values are described in the main text.

We therefore examined PPF in dorsal and ventral hippocampi in slices from WT and KO mice. As depicted in Figure 2, analysis of the changes in the relative amplitudes (Figure 2A) and field slope ratios (Figure 2B) measured at all the different interpulse intervals examined (see also Material and Methods) showed significant differences between the groups indicating a marked impact of the miRNA-132/212 gene deletion on the properties of dorsal vs. ventral responses during short-term synaptic plasticity. Mixed-effects model three-way ANOVA with Tukey multiple comparisons correction showed significant (**) effect of the interpulse interval (*p* = 0.0012), as well as highly significant (****) difference for WT dorsal–WT ventral vs. KO dorsal–KO ventral (*p* < 0.0001) and high significance (****) for WT dorsal–KO dorsal vs. WT ventral–KO ventral (*p* < 0.0001) for the analyses of the data from the raw amplitudes (with Chi-square = 20.98; df = 1; *p* < 0.0001; (****)) (Figure 2A). Accordingly, mixed-effects model three-way ANOVA with Tukey multiple comparisons correction showed significant (**) effect of the interpulse interval (*p* = 0.0033), as well as both significant (*) difference for WT dorsal–WT ventral vs. KO dorsal–KO ventral (*p* = 0.0327) and high significance (****) for WT dorsal–KO dorsal vs. WT ventral–KO ventral (*p* = 0.0001) for the analyses of the slopes (Chi-square = 14.71; df = 1; *p* = 0.0001; (***)) (Figure 2B).

### 2.3. miRNAs-132/212 Regulate the Region-Specific Effects of Corticosterone on Metaplasticity in the Mouse Hippocampus

**Figure 3:**
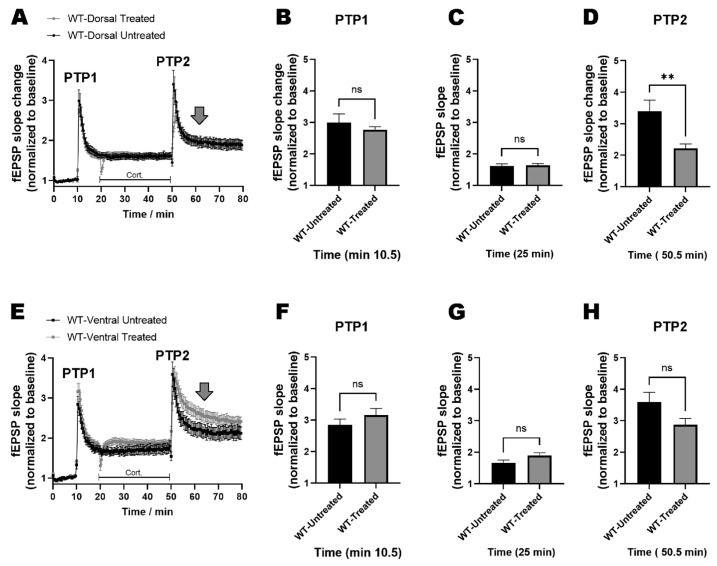
Corticosterone affects hippocampal metaplasticity in a region-specific manner. (**A**) Double high-frequency stimulation protocols, known to induce LTP (see also Materials and Methods) and generate robust peaks of post-synaptic-potentiation (PTP) responses (PTP1 and PTP2), were implemented in slices in order to examine the effect of 1 µM corticosterone on facilitated synaptic transmission and plasticity in the dorsal hippocampus of WT animals. No salient differences were apparent for the PTP1 response or the succeeding development of synaptic transmission during the exposure to corticosterone, as compared to the untreated control group. However, a very pronounced, statistically significant reduction of the PTP2 response was observed in the corticosterone-treated group, with the following field responses developing analogously to those of the untreated control group. Independent statistical analyses (details in the main text) were conducted for the PTP1 response measured at 10.5 min (**B**), which showed no significant differences; as well as for the development of synaptic transmission in the presence of corticosterone at 25 min (**C**), also showing no differences; and for the PTP2 response at 50.5 min (**D**), which showed significant (**) differences (statistical values in the main text). Corresponding analyses were done for the ventral hippocampus, which showed no differences for PTP1 and PTP2 (**E**), and also no significant differences in the field responses of 10.5 min (**F**), 25 min (**G**) and 50.5 min (**H**). Gray-filled large arrows show effects on metaplasticity. ns = not significant. *p* < 0.05 was considered significant. ** *p* < 0.01. Data are shown as mean ± SEM. Statistical values and the number of subjects are described in the main text.

We next analyzed the effect of the 1 µM corticosterone treatment on dorsal hippocampal metaplasticity in slices obtained from WT animals. To this aim, two consecutive LTP-inducing stimulation protocols known to induce metaplasticity [36,77] were delivered before and after corticosterone treatment, which generated the transient post-tetanic potentiation peaks PTP1 and PTP2 (see also Materials and Methods). Figure 3A shows the development in time of the averaged field-slope responses (normalized to the initial baseline). No differences between the untreated and the corticosterone-treated group were apparent for PTP1, and in the treated group, corticosterone treatment did not result in salient differences in the subsequent development of the potentiated field responses compared to the untreated group. However, the PTP2 response obtained after corticosterone treatment presented a statistically significant reduction in its initial amplitude, whereas the subsequent field slopes developed in time in a pattern that did resemble that of the untreated group (Figure 3A). Two-way RM-ANOVA with Bonferroni´s and Geisser–Greenhouse´s correction, and alpha set to 0.05 (with *n* = 10–11 animals per group), showed no significance for the effect of the treatment (F _(1, 19)_ = 0.01610; *p* = 0.9004) but a significant (****) time × treatment interaction (F _(159, 3021)_ = 2.482; *p* < 0.0001). Unpaired *t*-test (two-tailed) was also used to determine significance for three individual times: 10.5 min, corresponding to the first PTP response (Figure 3B); 25 min, corresponding to the early effect of corticosterone on the synaptic responses (Figure 3C); and 50.5 min, corresponding to the second PTP response (Figure 3D). Significant differences were found only for the PTP2 response (50.5 min: *p* = 0.0078; Welch-correction t = 3.149, df = 12.80; 10.5 min: *p* = 0.4654; Welch-correction t = 0.7529, df = 12.50; 25 min: *p* = 0.7769, Welch-correction t = 0.2876, df = 18.25).

An equivalent examination was conducted in order to study the effects of corticosterone on ventral hippocampal metaplasticity using the double LTP-inducing protocol in the WT group. As depicted in Figure 3E, the PTP1 responses showed no differences when the untreated and corticosterone-treated groups were compared. Two-way RM-ANOVA with Bonferroni´s and Geisser–Greenhouse´s correction, and alpha set to 0.05 (with *n* = 10–11 per group), showed no significance for the effect of the treatment (F _(1, 19)_ = 2.375; *p* = 0.1398) but a significant (****) time × treatment interaction (F (159, 3021) = 2.884; *p* < 0.0001). As conducted for dorsal hippocampal slices, unpaired *t*-test (two-tailed) was also used to examine differences in the ventral hippocampus for 10.5 min, 25 min and 50.5 min. As illustrated in Figure 3F–H, no significant differences were found in any of the three examined time points. (10.5 min: *p* = 0.2748; Welch-correction t = 1.124, df = 19; 25 min: *p* = 0.0814, Welch-correction t = 1.840, df = 19; 50.5 min: *p* = 0.0703; Welch-correction t = 1.917, df = 19), but a mixed-effects model (REML) without sphericity assumption, used to independently verify the behavior of the data right after the PTP2 (min 52–79), found a highly significant time × treatment interaction (*p* < 0.0001 (****), F _(54, 1026)_ = 1.959), a phenomenon not observed in dorsal hippocampal slices (see large down-pointing gray-filled arrows after PTP2 in Figure 3A,E).

### 2.4. miRNA–132/212 Gene Deletion Disrupts the Region-Specific Effect of Corticosterone on Hippocampal Metaplasticity

**Figure 4:**
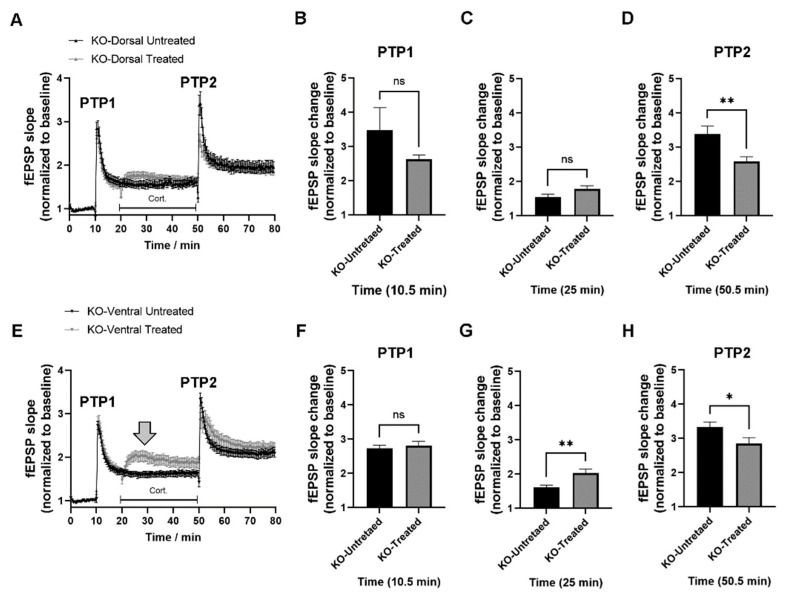
miRNAs–132/212 gene deletion impairs the region-selective effect of corticosterone on hippocampal synaptic plasticity. The properties of synaptic transmission and plasticity examined in the dorsal hippocampus of untreated and corticosterone-treated slices derived from miR–132/212^−/−^ mice were comparable with the WT group described above. That is, no major differences were observed for PTP1, but a significant reduction in the PTP2 response (**A**) was apparent, also with no detectable differences for 10.5 min (**B**) and 25 min (**C**), and a statistically significant (**) difference between untreated and treated groups for 50.5 min (**D**). However, for the ventral hippocampus, while slices from miR–132/212^−/−^ mice exhibited similar PTP1 responses for the untreated and corticosterone-treated groups (**E**), the facilitated postsynaptic responses measured in the presence of corticosterone presented an enhanced amplitude (large gray-filled arrow) as well as a significant reduction of the PTP2 response, to a degree that was not observed in the corticosterone-treated WT group. Statistical analyses conducted for responses measured at 10.5 min showed no significant differences (**F**); whereas both recordings at 25 min (**G**) and 50.5 min (**H**) showed statistically significant differences (** and *, respectively). ns = not significant. * *p* < 0.05, ** *p* < 0.01. Data are shown as mean ± SEM. Statistical values and number of subjects are described in the main text.

In order to assess the relevance of the microRNAs 132 and 212 as regulators of the effects of corticosterone in hippocampal synaptic transmission and metaplasticity, we next investigated the acute impact of corticosterone stimulation on the dorsal and ventral hippocampus using slices from miR–132/212^−/−^ mice. We first examined the effects of corticosterone on metaplasticity using the paired LTP-inducing protocol in the dorsal hippocampus. As shown in Figure 4A, slices from miR–132/212^−/−^ mice presented with metaplasticity responses are indistinguishable from those observed in slices from their related WT counterparts after corticosterone treatment. That is, corticosterone induced a reduction in the amplitude of the PTP2 response in miR–132/212^−/−^ slices but did not affect the temporal development of the subsequent field responses relative to the untreated miR–132/212^−/−^ control group. Two-way RM-ANOVA with Bonferroni´s and Geisser–Greenhouse´s correction, and alpha set to 0.05 (with *n* = 10–11 per group), showed no significance for the effect of the treatment (F _(1, 19)_ = 0.002677; *p* = 0.9593) but a significant (****) time × treatment interaction (F _(159, 3021)_ = 5.025; *p* < 0.0001). Unpaired *t*-test (two-tailed), used to examine differences at 10.5, 25 and 50.5 min, returned non-significant values of *p* = 0.2252 and Welch-correction t = 1.280 with df = 11.75 for 10.5 min (Figure 4B); *p* = 0.0549 and Welch-correction t = 2.048 with df = 18.62 for 25 min (Figure 4C); and significant values of *p* = 0.0083 (****) with Welch-correction t = 3.011 and df = 16.01 only for 50.5 min (Figure 4C).

We subsequently analyzed the impact of corticosterone on metaplasticity in the ventral hippocampus of slices derived from miR–132/212^−/−^ mice. Figure 4E shows comparable metaplasticity-related LTP responses between the untreated and the corticosterone-treated groups for the PTP1 response and no statistically significant differences at 10.5 min timepoint (Figure 4F). However, corticosterone produced a statistically significant increase in the synaptic responses in the presence of corticosterone as recorded at 25 min, which is indicated by a large gray-filled down-pointing arrow in Figure 4E, a phenotype also revealed at the 25 min timepoint in Figure 4G (**). Moreover, corticosterone also produced a significant reduction in the PTP2 response in ventral hippocampal slices from miR–132/212^−/−^ mice (Figure 4H), an effect not detected in corticosterone-treated ventral hippocampal slices derived from WT mice (Figure 3H). Two-way RM ANOVA with stacked matching and no sphericity assumption (done with Geisser–Greenhouse´s and Bonferroni´s corrections and alpha set to 0.05) for data depicted in Figure 4E reported no significance for the effect of treatment (*p* = 0.0991) and a highly statistically significant (****) time × treatment interaction (*p* < 0.0001; F _(159, 3180)_ = 3.899). Independent two-tailed unpaired *t*-test yielded values of *p* = 0.6237 with Welch-corrections of t = 0.4998 and df = 16.76 for datapoints at 10.5 min (Figure 4F); *p* = 0.0064 (**) with Welch-corrections of t = 3.232 and df = 13.26 for 25 min (Figure 4G); and *p* = 0.0492 (*) with Welch-corrections of t = 2.107 and df = 18.27 for 50.5 min (Figure 4H).

Taken together, all these observations point towards a possible involvement of the miRNA 132/212 cluster family in the region-specific regulation of the effects of glucocorticoid hormones on hippocampal synaptic metaplasticity functions and memory-related emotional behaviors. These data further suggested that molecular elements known to mediate in the regulation of both synaptic functions and glucocorticoid hormone activity could be differentially affected by corticosterone in WT and miR–132/212^−/−^ mice hippocampi. In an effort to launch a first experimental verification of this hypothesis, we used the technique of western blot (Materials and Methods) and examined the expression levels of CREB, Sirt1, MSK1, CDK5 and PTEN in the hippocampi of WT and miR–132/212^−/−^ mice.

### 2.5. Deletion of miR–132/212^−/−^ Prevents Enhanced Expression of CREB in the Hippocampi of Corticosterone Treated Slices

The participation of CREB in the regulation of synaptic plasticity and hippocampus-dependent learning and memory functions has been extensively described, and functional crosslinks between microRNA-mediated regulation (including the 132/212 cluster) and CREB expression have also been proposed [44,78,79,80,81,82,83]. Moreover, our group has recently described significantly enhanced levels of CREB in the hippocampus of miR–132/212^−/−^ mice [61]. However, to the best of our knowledge, the acute effects of corticosterone on the levels of CREB has not been examined in hippocampi lacking miR-132/212. We therefore prepared acutely-dissociated hippocampal slices from both WT and miR–132/212^−/−^ mice and subsequently stimulated them for 1 h with corticosterone. Two-way ANOVA (Alpha 0.05) reported a significant effect of the genotype (F (1, 12) = 6.504; *p* = 0.0255), high significance for the effects of the treatment (F _(1, 12)_ = 45.16; *p* < 0.0001), and a significant interaction between factors (F _(1, 12)_ = 80.05; *p* < 0.0001).

**Figure 5:**
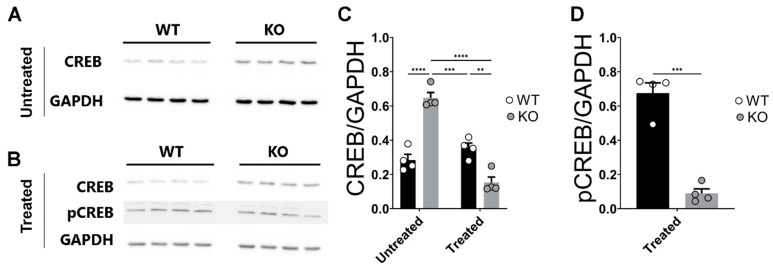
Corticosterone induces reduction in the levels of CREB in miR–132/212^−/−^ mice hippocampus. (**A**,**B**) Representative photographs of blotting membranes containing transferred proteins obtained from untreated and corticosterone-treated hippocampal tissue from WT and miRNA-132/212^−/−^ (KO) mice, respectively. The membranes were incubated with antibodies for CREB, pCREB and GAPDH. (**C**) The chart shows the averaged data for the levels of CREB normalized to those of the GAPDH as derived from densitometric analysis of the blots. The levels of CREB are significantly augmented in the hippocampus of the miRNA-132/212 KO mice and significantly reduced in response to corticosterone treatment. (**D**) Charts of the blots averaged densitometry data for the levels of pCREB relative to GAPDH, as examined using corticosterone-treated hippocampal tissue from WT and miRNA-132/212^−/−^ mice. Note the significant reduction in the levels of the two forms of CREB in response to corticosterone. The results in all charts are shown in each case as a fold change relative to the detected levels of the enzyme GAPDH. ** *p* < 0.01, *** *p* < 0.001, **** *p* < 0.0001. Data are shown as mean ± SEM (*n* = 4 animals per group).

As shown in Figure 5A,C, further post hoc analyses revealed that untreated hippocampi derived from miR–132/212^−/−^ mice presented enhanced expression levels of CREB compared to untreated WT controls, thus corroborating our previously reported observations [61]. However, both the levels of CREB (Figure 5B,C) were considerably reduced in the corticosterone-treated hippocampi of miR–132/212^−/−^ mice when compared to both untreated KO miR–132/212^−/−^ slices and corticosterone-treated hippocampi from the WT animals. Phospho-CREB was also downregulated in KO compared to WT slices when treated with corticosterone (Figure 5B,D), as shown by unpaired *t*-test (*p* < 0.0001; t = 8.834, df = 6).

### 2.6. Sirt1 Protein Levels Are Enhanced and Insensitive to Corticosterone Stimulation in the miR–132/212^−/−^ Mice Hippocampi

**Figure 6:**
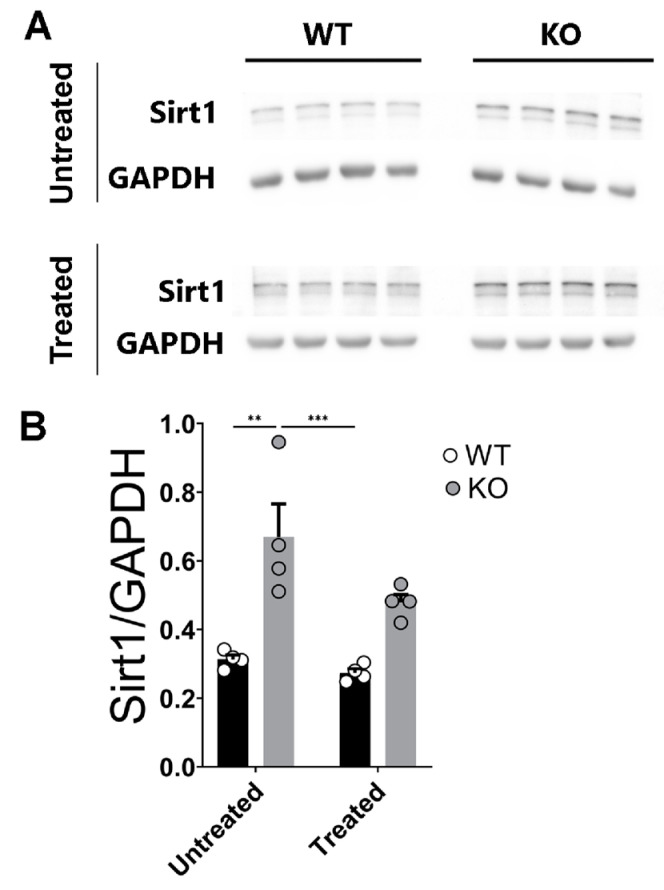
Corticosterone does not affect the otherwise enhanced levels of Sirt1 detected in the miR–132/212^−/−^ mice hippocampus. (**A**) Representative photographs of blot membranes derived from WB studies using untreated (upper set) and corticosterone-treated (lower set) hippocampal tissue from WT and miRNA-132/212^−/−^ (KO) mice. The membranes were incubated with antibodies for Sirt1 and GAPDH. (**B**) Averaged levels of Sirt1 under untreated and corticosterone-treated conditions, normalized to GAPDH values, as derived from densitometric analysis of blots. The levels of Sirt1 appeared significantly enhanced in the untreated hippocampi from miRNA-132/212^−/−^ (KO) mice. The levels of hippocampal Sirt1 continued to be significantly enhanced upon corticosterone-treatment in miRNA-132/212^−/−^ (KO) mice compared to those of the WT controls. Results in charts represent fold changes relative to GAPDH. ** *p* < 0.01, *** *p* < 0.001. Data are shown as mean ± SEM (*n* = 4 animals per group).

A functional link has been very recently established between the brain neuronal microRNAs miR-132 and miR-212 and their target-regulated protein Sirtuin 1 (Silent Information Regulator 1; in short, Sirt1) in the context of Alzheimer’s Disease [84]. Sirt1 has indeed been shown to regulate endocrine activity and participates in the regulation of memory-related neuronal functions through its capability to induce axonal and dendritic morphological rearrangements [84,85], see also [86]. Moreover, corticosterone (the brain stress-signaling molecule in rodents equivalent to cortisol in humans) has also been shown to dose-dependently regulate the levels of Sirt1 in cellular stress models [87]. While a crosslink between Sirt1 and the miRNAs 132/-212 had been previously examined [88], the acute impact of corticosterone on the levels of hippocampal Sirt1 and its relation to the levels of the miRNAs 132 and 212 remained, to our knowledge, uncharacterized. We, therefore, next sought out to examine the acute effects of corticosterone on the levels of Sirt1 in the hippocampi of both WT and miR–132/212^−/−^ mice (see also Materials and Methods). Two-way ANOVA (Alpha 0.05) reported statistically significant differences (***) for genotype (F _(1, 12)_ = 31.52; *p* = 0.0001); also, significance (*) for treatment (F _(1, 12)_ = 5.293; *p* = 0.0401) with no differences for interactions (F _(1, 12)_ = 2.287; *p* = 0.1564). Subsequent post hoc analysis showed that the hippocampi of miR–132/212^−/−^ mice presented a significant enhancement in the levels of Sirt1, while the corticosterone treatment did not significantly revert this phenotype (Figure 6).

### 2.7. Corticosterone Effects on MSK1 Levels in WT and miR–132/212^−/−^ Mice Hippocampi

**Figure 7:**
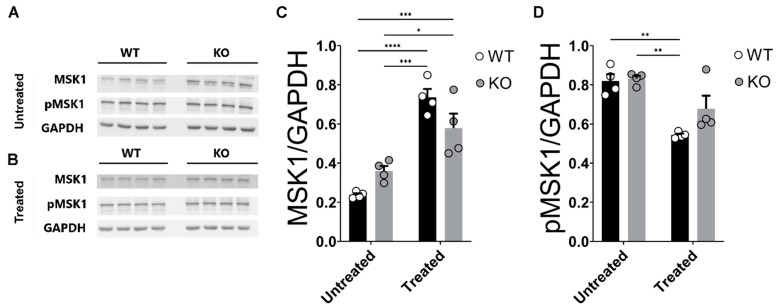
The levels of hippocampal MSK1 in WT and miR–132/212^−/−^ are comparable and enhanced by corticosterone. (**A**,**B**) Representative blots for untreated and corticosterone-treated hippocampal tissue from WT and miRNA-132/212^−/−^ (KO, in the figure) mice in membranes incubated with antibodies for MSK1, phospho-MSK1 (pMSK1) and GAPDH. (**C**) Averaged MSK1 levels, relative to GAPDH, as from densitometric analysis of blots. WT and miRNA-132/212^−/−^ presented with enhanced MSK1 levels in the corticosterone-treated groups. (**D**) Averaged pMSK1 levels, relative to GAPDH, as from densitometric analysis of blots. While the levels of pMSK1 were comparable between miRNA-132/212^−/−^ and WT hippocampi, corticosterone significantly reduced the levels of pMSK1 in the WT group. *p* < 0.05 was considered significant. * *p* < 0.05, ** *p* < 0.01, *** *p* < 0.001, **** *p* < 0.0001. Results represent fold changes relative to GAPDH. Data are shown as mean ± SEM (*n* = 4 animals per group).

The Mitogen- and Stress-activated Kinase 1 (MSK1) enzyme is a nuclear serine/threonine protein kinase whose in vivo activity becomes triggered upon activation of either the Mitogen-Activated Protein Kinases (MAPK) ERK1/2 or p38, which participate in the structural/functional modifications of the histones during the regulation of gene transcription that is associated with emotional processing and hippocampus-dependent learning and memory functions [89,90,91,92,93]. Moreover, it has also been shown that MSK1 is associated with the regulation of transcription of miR-132/212 [56]. However, how glucocorticoid stimulation could impact the levels of MSK1 in a miRNA-132/212^−/−^-dependent manner remained unknown. We therefore investigated the effects of acute corticosterone stimulation on the hippocampal levels of MSK1 in WT and miR–132/212^−/−^ mice. For total MSK1 analyses, two-way ANOVA (Alpha 0.05) reported no differences for genotype (F _(1, 12)_ = 0.1503; *p* = 0.7050), significant (****) differences for the treatment (F _(1, 12)_ = 63.25; *p* < 0.0001) and significant (**) differences for the interaction (F _(1, 12)_ = 9.618; *p* = 0.0092). For pMSK1 analyses, two-way ANOVA (Alpha 0.05) reported no significant differences for genotype (F _(1, 12)_ = 3.486; *p* = 0.0865), significant (***) differences for the treatment (F _(1, 12)_ = 30.38; *p* = 0.0001) and no significant differences for the interaction (F _(1, 12)_ = 2.473; *p* = 0.1418). Further post hoc analysis revealed that the untreated hippocampi from miR–132/212^−/−^ and WT showed comparable levels of MSK1 and phospho-MSK1 (pMSK1) (Figure 7A–D). Additionally, the corticosterone treatment resulted in an overall pronounced elevation in the detected levels of MSK1 in the hippocampi of both WT and miR–132/212^−/−^ animals, indicating that corticosterone has the ability to modulate the levels of total MSK1 in a manner independent of the levels of miRNAs 132/212 (Figure 7C). However, an inhibitory effect of corticosterone was observed for the levels of p-MSK1 in WT hippocampi that was not present in the hippocampi from miR–132/212^−/−^ mice (Figure 7D), indicating the possible role of pMSK1 in the corticosterone response that is missing in the KO mouse.

### 2.8. Unaltered Levels of CDK5 and PTEN in the Hippocampi of miRNA-132/212^−/−^ Mice

**Figure 8:**
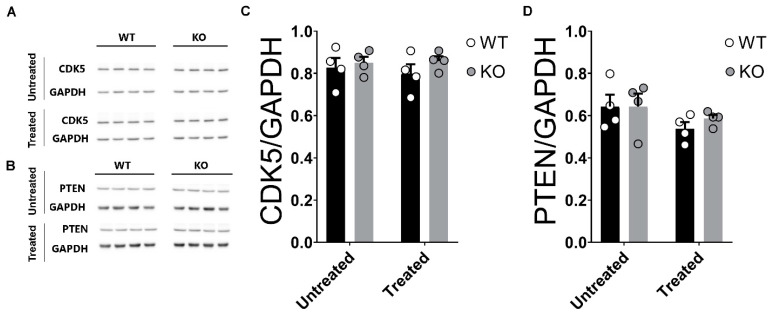
Corticosterone does not affect the otherwise comparable levels of CDK5 or PTEN in WT and miRNA-132/212^−/−^ mice hippocampi. (**A**,**B**) Blots for untreated and corticosterone-treated (treated) hippocampal tissue from WT and miRNA-132/212^−/−^ (KO) mice in membranes incubated with antibodies for CDK5, PTEN and GAPDH. (**C**,**D**) Averaged CDK5 and PTEN levels, respectively, relative to GAPDH, as from densitometric analysis of blots for untreated and corticosterone-treated tissue. No significant differences in the levels of CDK5 or PTEN were found between the untreated or corticosterone-treated hippocampi of WT and miRNA-132/212^−/−^ mice. Results represent fold changes relative to GAPDH. Data are shown as mean ± SEM (*n* = 4 animals per group).

Both CDK5 and PTEN are brain neuronal proteins expressed in the hippocampus that are involved in the regulation of memory-related synaptic functions [94,95,96,97,98,99,100,101,102,103]. Additionally, both CDK5 and PTEN have been also associated with the stress-related effects of corticosterone and the hippocampal function (see also [104,105,106] and [94,107,108], respectively). Similarly, both CDK5 and PTEN have been shown to be regulated by microRNAs (see e.g., [109,110] and [111,112], respectively). However, the impact of corticosterone on the hippocampal levels of CDK5 and PTEN and its relation to miRNAs 132 and 212 remained, to our knowledge, uncharacterized. We therefore examined the impact of corticosterone on the levels of both CDK5 and PTEN in the hippocampus of WT and miRNA-132/212^−/−^. Figure 8 shows data from WB experiments indicating that neither the deletion of the genes encoding for the miRNAs 132 and 212 nor the treatment with corticosterone affects the expression levels of CDK5 or PTEN in either WT or miRNA-132/212^−/−^ mice hippocampi. For CDK5 analyses, two-way ANOVA (Alpha 0.05) reported no significant differences for genotype (F _(1, 12)_ = 1.303; *p* = 0.2759), interaction (F _(1, 12)_ = 0.2628; *p* = 0.6175) or treatment (F _(1, 12)_ = 0.08639; *p* = 0.7738). For PTEN analyses, two-way ANOVA (Alpha 0.05) reported no significant differences for genotype (F _(1, 12)_ = 0.2911; *p* = 0.5994), no differences for the treatment (F _(1, 12)_ = 3.248; *p* = 0.0967) and no differences for the interaction (F _(1, 12)_ = 0.2734; *p* = 0.6106).

### 2.9. Reduced Anxiety-Like Behavior in miRNA-132/212^−/−^ Mice

**Figure 9:**
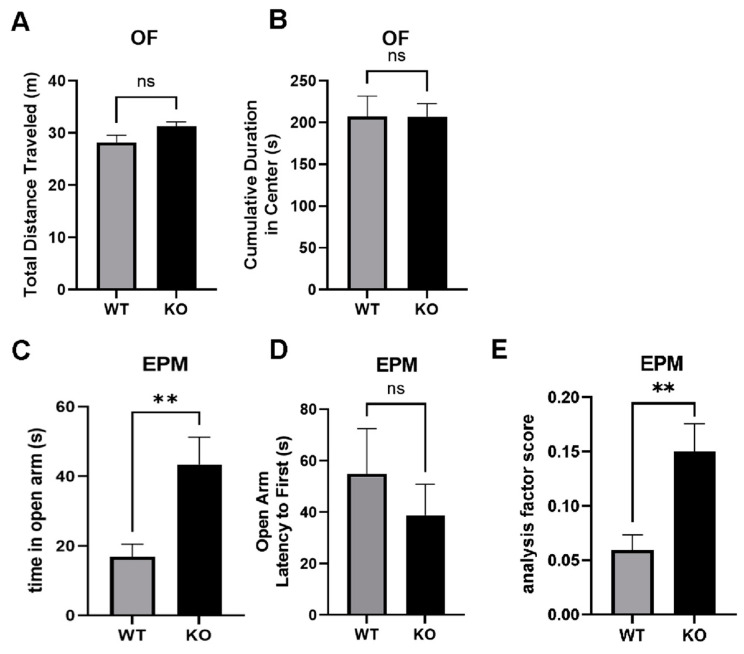
Performance of WT and miRNA-132/212^−/−^ mice in the open field (OF) and EPM. In the OF, no differences in the behavior of the animals were apparent after examinations of the total distance travelled (**A**) and cumulative time duration that the animals spent in the center (**B**). An *n* = 12 animals per group was used for the OF studies. In the EPM, miRNA-132/212^−/−^ (KO) mice presented significantly increased time spent in the open arms (**C**), whereas no differences were detectable in the latency to the first enter to the open arms (**D**) compared to their WT littermates used as controls. (**E**) Analysis of the time spent in the open arms relative to closed arms showed enhanced time in the open arms for miRNA-132/212^−/−^ mice compared to WT controls. An *n* = 10–12 animals per group was used for EPM studies, ns = not significantly different. *p* < 0.05 was considered significant. ** *p* < 0.01. Data are expressed as mean ± SEM.

Our data from the pharmacological treatment with corticosterone in hippocampal electrophysiology, as well as the western blot analyses of changes in the levels of molecules involved in the physiological responses to stress described here in response to corticosterone treatment, suggested that the miRNA 132/212 cluster was implicated in the regulation of emotional behaviors. Consequently, changes in anxiety-like behaviors were predicted to be detectable in miRNA-132/212^−/−^ mice. In order to test this hypothesis, we conducted behavioral experiments using the open field test (OFT) and the elevated plus maze [EPM (Materials and Methods)]. As shown in Figure 9, no significant differences in the behavior of WT and miRNA-132/212^−/−^ mice were observed in the OFT. In the OFT, unpaired two-tailed *t*-test yielded values of *p* = 0.064 (t = 1.949, df = 22) for total distance traveled comparisons (Figure 9A) with no between-group differences found in the cumulative duration in the center of the open field (Figure 9B). In the EPM, however, statistically significant differences in features associated to anxiety-like behaviours became apparent in miRNA-132/212^−/−^ subjects when compared to WT mice. Unpaired two-tailed *t*-test yielded values of *p* = 0.0094 (t = 2.874, df = 20) for the absolute time spent in the open arm (Figure 9C), with no differences detected for the latency to the first access to the open arm (*p* = 0.4491; t = 0.7721, df = 20) in the EPM (Figure 9D). Moreover, when the time spent in the open arms was analysed relative to the time spent in the closed arms (here referred to as analysis factor score), miRNA-132/212^−/−^ animals exhibited significantly (**) enhanced time spent in the open arms compared to their relative WT control counterparts (*p* = 0.0094; t = 2.872, df = 20) (Figure 9E). Taken together, all these observations encourage further research in order to independently verify the herein proposed potential involvement of the 132 and 212 miRNA cluster in the modulation of stress-related emotional responses mediated by steroid hormones belonging to the pituitary adrenocortical axis.

## 3. Discussion

Steroid-hormones, including cortisol, have been proposed as modulators of the hippocampal functions in the context of mood-related disorders in humans [113,114]. Corticosterone is a very potent steroid-hormone influencing several neuronal functions (including neuronal morphology and synaptic plasticity in rodents), and also affecting learning and memory and emotional behaviors in a manner analogue to that of human cortisol [16,17,18,19]. In humans, the physiological relevance of neurohormonal regulators becomes even more apparent for its relation to conditions such as stress, depression and anxiety; and for its crosslink with other maladies and psychiatric disorders, some of which have been widely studied in experimental animal models [6,7,8,9,10,11,12,13,14]. In the amygdala, a brain region critical for the regulation of learning, memory, and emotional behaviors [115,116], regulation by steroid-hormone-mediated signaling has been previously described [63,117,118,119].

The hippocampus had also been shown to play pivotal roles not only in spatial learning and memory, but also in the processing of emotion-related memory storage [23,24,25,26,27,28,29,120]. Circa 123 years ago, Ramon y Cajal had indeed provided some of the first anatomical, pictographic renditions highlighting some of the distinctive morphological differences between the dorsal and the ventral hippocampus (see also [121]). However, experimental/unequivocal demonstrations about the relevance of specific regions of the hippocampus as selected areas important for the regulation of emotional behaviors started to emerge only about 15-20 years ago [121,122,123,124,125,126,127], and, notwithstanding of this, little continue to be known about the molecular mechanisms determining the region-specific effects of steroid-hormones on the hippocampal function [30,31,32,33].

### 3.1. miRNAs 132/212 in the Brain Neuronal Function

Using a mouse model of learned safety, Ronovsky et. al., proposed that, in the amygdala, miR-132/212 regulate fear-inhibitory mechanisms as well as emotional-responses and plasticity-related synaptic functions [62]. Ronovsky et. al., also identified candidate proteins in the amygdala as potential gene targets for miRNA-132/212 which could participate in the miR-132/212-mediated regulation of mood-related behaviors [62]. We here expanded that line of research, by addressing the impact of miRNA-132/212 gene deletion on hippocampal synaptic functions and the effects to emotion-related steroid-hormone stimulation. Our data proposes the first functional description, to the best of our knowledge, for an involvement of miR-132/212 in the region-specific regulation of the effects of steroid-hormones on synaptic transmission and plasticity in the hippocampus.

Interestingly, other authors have found a long-term enhancement of corticosterone as well as impaired hippocampal synaptic plasticity in trauma-susceptible mice [128]. In line with these studies, and in agreement with our data, other reports have also shown a dysregulation of miRNAs in the hippocampus of trauma-susceptible mice [129]. Our behavioral examinations showing altered anxiety-like behavior in miRNA-132/212^−/−^ animals provide further support to the possible involvement of the miRNAs 132/212 in mood-related behaviors, as they reveal the existence of basal differences in behavioral responses that are known to be influenced by neuroendocrine signaling. Discrepancies between data from different tests examining anxiety-like behaviors (e.g., using the open field) have precedents in the scientific literature. These differences can be due to factors such as the size of the experimental arenas (many different laboratories use open fields of different dimensions), differences in the different intensities of illumination used in different laboratories, or the use of partially reflective or even transparent materials in some arenas (see e.g., [130]). All these different subtle factors are known to influence the outcome in different experimental settings depending on different animal strains; depending on the type of pharmacological treatments; or depending on the genetic modifications introduced into the experimental subjects (see also [131,132,133,134,135,136,137]). Consequently, additional examinations (implementing other behavioral tests (e.g., Novelty suppressed feeding; Light/dark box, or Social Interaction Tests), are therefore encouraged.

### 3.2. Proteins Associated with Mood and Steroid Hormone Signaling

We here provide a basic biochemical analysis of proteins implicated in the regulation of steroid-hormone signaling, which propose the potential participation of the miRNAs 132/212 in the regulation of hippocampal steroid hormone signaling and emotion-related behaviors. Our results are in line with previous observations from our group and others [53,112], which pointed towards a possible involvement of miRNA-132/212 in the processing of emotion-related functions. We had previously described that miRNA-132/212 deletion influences the expression levels of alpha7-nAChR hippocampal receptors [43], which, in the basolateral amygdala, are proposed to mediate emotional behaviors [138,139]. We had also described that miRNA-132/212 deletion influenced the effects of nicotine (a modulator of mood and anxiety [140]) on hippocampal synaptic plasticity [61]. Previous reports had also associated the function of SIRT1 with miR-132- and miR-212-mediated regulation in the context of aging and Alzheimer’s Disease [84]. Moreover, the levels of miR-132 and miR-212 have been shown to become primarily augmented in the hippocampus in response to short-term (5 h) but not to long-lasting (15 days) exposure to stress, and depletion of the miR-132 has been shown to result in enhanced anxiety-like behaviors [112]. In this last report ([112]), the authors used single miR-132 and combined miRNA-132/212 depletion and showed, in both cases, altered expression levels for hippocampal SIRT1 and PTEN-2; two proteins known as targets of miR-132 that are involved in the modulation of anxiety-like behaviors. Interestingly, while the pioneering results from Aten, et.al., 2019 [112] already showed a marked, yet not significant, trend towards enhanced levels of SIRT1 in miRNA-132/212^−/−^ mice hippocampi, their results are perfectly in line with our observations here showing (with different methods) significantly enhanced levels of SIRT1 in the hippocampus of miRNA-132/212^−/−^ mice. Taken together, data derived from the pioneering studies from the group of Dr. Obrietan [112], together with our data here describing enhanced levels of SIRT1 in the hippocampi of miRNA-132/212^−/−^ mice (as compared to their related WT counterparts), and also the absence of significant effects of corticosterone in the two groups showed by us, indicate that whereas the presence of miRNAs 132/212 might negatively regulate the levels of SIRT1, this action is not affected by corticosterone treatment.

Additionally, previous reports have proposed a role for the proteins CDK5 and PTEN in the regulation of brain neuronal synaptic functions. For example, PTEN has been associated to the regulation of the cognitive function (including social behaviors) likely via its capability to regulate brain growth and to modulate neuronal circuit formation and synaptic functions, as examined in cortico-amygdala synapses [94]. Other groups have established PTEN is involved in the regulation of emotion-related fear memories as well as spatial memory, and that PTEN is also a critical regulator of hippocampal LTP via its functional association with the protein CaMKII [95] (see also [96]). CDK5, on the other hand, has been functionally involved in the regulation of hippocampal dendrite morphology [101], in the regulation of both hippocampal neurotransmitter release and amplitude of hippocampal field EPSP slope [102], and CDK5 has been also proposed as a possible molecular regulator of amyloid beta production and in the mechanisms associated to the pathogenesis of Alzheimer’s disease [98,103].

Our collaborative groups had also described PTEN as a potential target for miRNAs 132 and 212 in the amygdala [62]. Moreover, previous reports have described that the enhancement in the levels of miR-132 can induce augmented expression of CDK5 [141]. However, our findings here show comparable levels of PTEN and of CDK5 in WT and miRNA-132/212^−/−^ mice hippocampi without detectable effects in both cases of corticosterone. These observations thus suggest that miRNA-132/212^−/−^ might not play major roles in the regulation of the levels of PTEN and CDK5 in the mouse hippocampus. Nevertheless, given that total hippocampal tissue was examined here, further studies need to be conducted examining, separately, whether differences in the levels of these proteins or their transcripts might still exist when dorsal vs. ventral hippocampal regions are examined and compared using higher resolution techniques.

Here, we also show that whereas MSK1 shows comparable levels in WT and miRNA-132/212^−/−^ hippocampi (and in both cases, their levels become strikingly enhanced by corticosterone), the levels of phospho-MSK1, on the contrary, become significantly reduced only in WT hippocampi. Interestingly, and in line with our results here, previous observations have proposed a BDNF-related regulation in the levels of the miRNA-132/212 clusters via MSK activity [56], all together thus accumulating experimental evidence in support for the existence of a possible functional hippocampal crosslink between miRNA-132/212^−/−^ and MSK1 likely associated to the regulation of the effects of steroid hormones and, possibly, associated emotional and behavioral effects.

**Figure 10:**
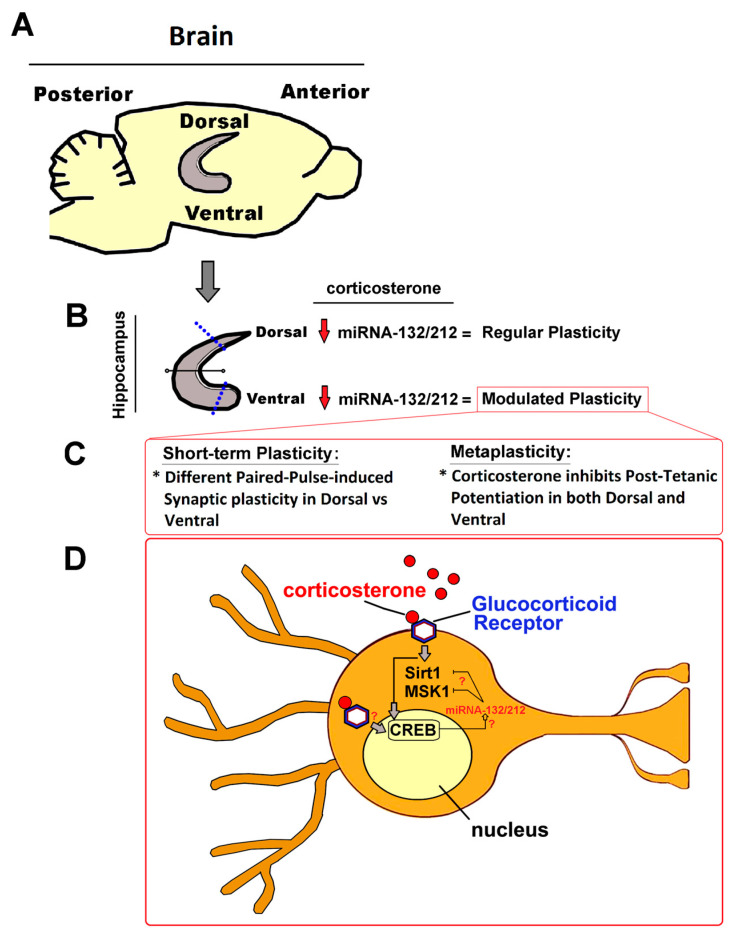
A possible role of the miRNA-132/212 family in the regulation of the effects of corticosterone on hippocampal synaptic plasticity. The hippocampus (**A**,**B**) is not a homogenous structure, as it presents several anatomical and functional differences between its dorsal and ventral regions. For example, the dorsal region has been shown to be primarily involved in spatial learning and memory functions, whereas the ventral region has been predominantly implicated in emotion-related processing. Here, we postulate that changes in the levels of miRNA-132/212 (e.g., its downregulation, as illustrated by red arrows in (**C**), could result in changes in the properties of both short-term and long-term forms of synaptic plasticity as well as in modulated metaplasticity (**C**). Alterations in the levels of miRNA-132/212 could also influence the synaptic responses to neuromodulators, such as corticosterone (**D**), which could, in a loop-like manner, impact the levels of miRNA-132/212 via activation of CREB, thus regulating the expression of proteins known to be downstream components of the glucocorticoid receptor (such as Sirt1 and MSK1). This work therefore proposes that miRNA-132/212 might contribute to increasing the in vivo mechanisms responsible for the functional heterogenicity between hippocampal regions, which might be critical to differentially distribute the effects of corticosterone on brain areas important for different cognitive functions.

Additionally, we observed significantly enhanced levels of hippocampal CREB in miRNA-132/212^−/−^ mice, together with a significantly stronger reduction of CREB levels in response to the corticosterone treatment (an effect of corticosterone also observed for pCREB) compared to WT mice hippocampi. Future research addressing the levels of pCREB under untreated condition might provide a larger picture of the impact of the miRNA 132/212 gene deletion on the hippocampal response to corticosterone and how this, comparatively, affects the levels of CREB phosphorylation (see also Figure 10). Abundant literature has linked CREB to hippocampal function in the context of stress ([92,119,142,143,144,145]) and anxiety ([80,146,147,148,149]). The link between CREB and members of the miRNA-132/-212 gene cluster has also been described before (see for example [150,151]). Similarly, an involvement of miR-132/212 in stress/anxiety-related behaviors as well as in the regulation of the levels of Sirt1 and PTEN have been also recently described [112]. Our data here showed that miRNA-132/212^−/−^ deletion significantly changes the effects of corticosterone on the levels of CREB, which is a critical regulator of memory [152], but not of other proteins involved in the responses to stress (e.g., PTEN).

Our work thus provides unprecedent data suggesting that miRNA-132/212 might participate, in vivo, in the regulation of synaptic plasticity and mood-related behaviors by fine-tuning the effects of steroid hormones via regulation of the levels of specific gene-product targets in an inter- and intra-brain-region selective manner (Figure 10). Further experiments are thus required in order to elucidate whether the levels of proteins that mediate in steroid hormone regulation are influenced by miRNA-132/212 in a subregion-specific manner. In the light of recent reports linking the hippocampus to learned safety [153,154], our findings also propose miRNA-132/212 as a potential modulator of learned safety through their capability to influence the levels of molecular targets responsive to neuroendocrine signals in both the hippocampus and in the amygdala (see also [62,155,156]). These observations encourage further verification in female subjects, as the existence of gender-specific effects of steroid-hormones on behavior, cognition, as well as on neuronal morphology and plasticity-related functions, have been established [157,158,159,160,161,162,163,164,165], and also brain microRNAs are regulated in a sex-dependent manner (see also [166,167,168]).

## 4. Materials and Methods

### 4.1. Animals

All the experiments reported here were performed using male adult (8–10 weeks old) wild type (WT) C57Bl/6 (substrain N) mice as well as knockout (KO) miRNA-132/212 (miRNA-132/212^−/−^) mice. These knockout mice, originally produced and described by the group of Dr. Pankratov [57], were engineered via the insertion of LoxP sites at non-coding regions (1st intron and exon 2) of the RNA gene encoding for the miRNAs 132 and 212, and generated in a C57Bl/6 background [57]. Here, as controls, only WT littermate animals (as verified by PCR-based genotyping), were used. All experiments followed ethic directives of the Bundesministerium für Wissenschaft und Forschung of Austria (BMWF-66.009/0200-WF/V/3b/2016). ARRIVE and U.K. Animals usage guidelines (Scientific Procedures Act, 1986 and associated guidelines, EU Directive 2010/63/EU for animal experiments) were implemented. Animals were kept in standard Thoren Plexiglas cages housed in a colony-room with a temperature of (22 ± 2) °C. Since animal grouping has been reported to reduce stress and aggression [169], in agreement with reports examining the importance of cage size for animal wellbeing [170], 3–5 mice were grouped together per Thoren Plexiglass cage (with cages of ≈ 22 × 31 × 16 cm). Cages were located in a Thoren mouse vent rack with maximizer (Thoren, Hazleton, PA, USA). The housing room had an automatically controlled illumination system programmed on a 12 h light/dark cycle (with light switched on at 6:00 a.m. to deliver (200 ± 20) lux). All cages were provided with aspen wood bedding (ABEDD-LAB & VET Service, Vienna, Austria), and every cage was equipped with 2 layers of fragrance-free TORK Advance Soft paper (Tork AT, Essity Austria GmbH, Storchengasse 1, Vienna (1150) Austria) which animals consistently shredded and used as nesting material. Both bedding and nesting materials were entirely changed once a week. All animals had both food and water available ad libitum. All the animals were handled by expert technical personnel, and an expert veterinarian supervised animal management. While in this work we are only including male animals, our group is also currently conducting parallel experiments using female experimental subjects in order to examine the possible influence of sex as a critical factor mediating the effects of both corticosterone treatment and miRNA-132/212 gene deletions on the biophysical properties of hippocampal circuits (see also Discussion).

### 4.2. Animal Grouping and Work Plan

Experimental animals were randomly assigned to one of the different experimental groups studied, which were organized as follows: 2 groups were used for untreated controls (untreated WT vs. untreated KO), and 2 groups were used for corticosterone-treated (corticosterone-treated WT vs. corticosterone-treated KO). For the electrophysiological studies (see below) and all the four groups referred, the experiments were organized by sub-dividing the recordings conducted in the hippocampus into recordings obtained from the dorsal and ventral regions. For the western blot analysis, the experimental groups studied were organized as follows: 2 groups were used as untreated controls (untreated WT vs. untreated KO), and 2 groups were used for corticosterone-treated (corticosterone-treated WT vs. corticosterone-treated KO). It must be noted that for the WB experiments, the entire hippocampus was used; that is, the hippocampal tissue was not sub-divided into dorsal or ventral regions (see also the sections Discussion and Limitations of this work). For behavioral studies, animals were separated into two major groups (WT vs. KO), and they were not subjected to any pharmacological treatments. The number of subjects used in each experiment has been noted in the main text and/or in Figures Legends.

### 4.3. Slice Electrophysiology

For the preparation of hippocampal slices, the animals were subjected to mild sedation with low CO_2_ inhalation followed by quick cervical dislocation and swift decapitation using a sharp-blade guillotining (DCAP-M, World Precision Instruments, Inc., Sarasota, FL, USA). Brains were extracted and placed on an ice-cold artificial Cerebrospinal Fluid (aCSF) solution containing (in mM): 125 NaCl, 2.5 CaCl_2_, 2.5 KCl, 1 MgCl_2_, 20 NaHCO_3_, 25 D-glucose, 1 NaH_2_PO_4_ (pH 7.4). Hippocampi were transversally sliced using a McIlwain tissue chopper (TC752, Campden Instruments Ltd., Loughborough, UK) into 400 μm thick sections. Slices were transferred into an aCSF-filled recovery chamber submerged in a bath filled with water at 30 °C, where they recovered for at least 1 h before electrophysiology measurements. All the aCSF solutions were continuously supplied with a carbogen gas mixture (95% medical O_2_ + 5% medical CO_2_). The slice electrophysiology measurements were conducted with slices placed in a submerged recording chamber that was continuously supplied with 3–4 mL/min of a pre-carbogenated aCSF that had been pre-warmed at (30 ± 2) °C. Field excitatory postsynaptic potentials (fEPSPs) were obtained through heat-pulled glass capillary pipettes (capillary glass from Harvard Apparatus, GmbH; Hugo Sachs Elektronik, Germany). Capillary tubes were pulled using a horizontal P-87 puller from Sutter Instrument (Model P-87, Novato, CA (94949), USA). Heat-pulled capillary pipettes were then back-filled with aCSF, yielding series resistances of (3 ± 1) MΩ. fEPSPs were registered from the hippocampal CA1 region, with recording electrodes positioned at the stratum–radiatum layer. fEPSPs were evoked by electrically stimulating the Schaffer´s collateral projections that originated from the CA3 region, as described before [43,68]. To induce the fEPSPs, biphasic-square pulses of voltage were delivered through bipolar electrodes made of tungsten wire isolated to the tip with a Teflon coating layer (~50 µm diameter tips). The voltage pulses were generated from an ISO-STIM 01D stimulator (NPI Electronics, Tamm, Germany).

To examine the properties of basal synaptic transmission, input/output (I/O) curves were generated by plotting the raw amplitudes (and first-decaying phase slopes (normalized to maximum)) of the recorded output fEPSPs against the different values of delivered input voltages, consisting of increasing voltage steps of 200 μs, with pulses of 0–9 V delivered (in 1 V increments) with interpulse intervals of 15 s (see Figure 1).

In order to induce long-term potentiation (LTP), 5 separate sets of electrical stimulation were applied (500 ms apart), each comprising a total of 10 biphasic voltage pulses (100 μs/phase) delivered at 100 Hz (Figure 1; see also [66,67,68,171,172]). For LTP experiments, 10 min baseline field recordings were followed by the delivery of the LTP-inducing protocol; subsequently, an additional 40 min of field recordings were obtained, and a second LTP-inducing electrical stimulation protocol was delivered, followed by 30 min of field recordings. This protocol, thus, comprises an experimental way to induce hippocampal metaplasticity (see also [36,77]). Changes across time in the values of the slopes of the initial field decay (obtained by offline linear fittings of the recorded traces and normalized to baseline) were used as a measure of synaptic plasticity. LTP measurements were averaged from the values obtained from all slices within each animal, thus generating a single value per subject. For the electrophysiological recordings, the corticosterone (1 μM) treatment was conducted only for LTP experiments as follows: 10 min of baseline recordings > 1st high-frequency stimulation step > 10 min of recordings in ACSF solution > 30 min recordings in either ACSF solution (for untreated controls) or ACSF solution + 1 μM corticosterone applied in the bath (for the treatment groups) > 2nd high-frequency stimulation step and immediately start completely washing out the bath solution using only ACSF solution without corticosterone for the rest of the recordings. Recordings were obtained using an AxoClamp-2B amplifier, digitalized using the Digidata-1440 interface, and acquired and analyzed using the pClamp-10 (version 11.1) software (all from Axon Instruments, Molecular Devices, 660-665 Eskdale Rd, Winnersh, Triangle, Wokingham RG41 5TS, UK). Paired-pulse-induced plasticity examinations were also conducted by delivering voltages evoking ~50% of the maximum inducible field amplitude, as described before [43].

### 4.4. Western Blotting

In order to conduct the Western blot (WB) examinations, hippocampi from both WT and miR–132/212^−/−^ mice (8–10 weeks old) were extracted and hippocampal slices prepared, allowed to recover for 1 h, and subsequently stimulated with 1 µM corticosterone or vehicle (Cat.Nr.27840, Sigma-Aldrich, Zimbagasse 5, Vienna (1140) Austria) for 1 h. Immediately after, the tissue was carefully transferred into Eppendorf^®^ tubes and snap-frozen in liquid nitrogen. For WB preparations, the tissue was treated with a freshly prepared homogenizing protein lysis buffer with the following composition (in mM): 150 NaCl, 1 EDTA, 10 Tris-HCl, 10 NaF, 10 Na_3_VO_4_, 5 Na_4_P_7_O_2_, 0.5% Triton ×100, 1% SDS and the protease inhibitor cocktail cOmplete™ (Roche Diagnostics, Mannheim, Germany). Samples of protein-containing tissue extracts were subsequently weight-separated by 10% SDS-PAGE gel electrophoresis. Separated protein contents were then transferred from the gels into polyvinylidene fluoride (PVDF) membranes, which were then subjected to 1 h of blocking treatment (5% BSA in TBST) at room temperature. The membranes were then exposed to an overnight bath treatment at 4 °C with the respective primary antibodies. The next day, the membranes were washed out and exposed to a 1 h bath treatment (room temperature) with the corresponding secondary antibodies. Indirect detection of the hippocampal protein levels was determined by chemiluminescence-reporter labeling of secondary antibodies and fluorescent image acquisition using a FluorChem HD2 system (Alpha Innotech, San Leandro, CA, USA). Obtained images were examined using the open-source image analysis software ImageJ (Version 1.53t) [173]. Protein levels from detected bands were quantified by densitometrical analysis with data normalized to values of GAPDH levels. The antibodies used were: CREB (Cell Signaling Technology (Danvers, MA, USA); USA, Cat. Nr. 9197); p-CREB (Cell Signaling Technology; USA, Cat. Nr. 9196s); Sirt1 (Cell Signaling Technology; USA, Cat. Nr. 2028); MSK1 (Cell Signaling Technology; USA, Cat. Nr. 3489); p-MSK1 (Cell Signaling Technology; USA, Cat. Nr. 9595); CDK5 (Cell Signaling Technology; USA, Cat. Nr. 2506); Pten (Abcam (Biomedical Campus, Discovery Dr, Trumpington, Cambridge CB2 0AX, United Kingdom); UK, Cat. Nr. ab154812); GAPDH (Thermo Fisher Scientific. 168 Third Avenue. Waltham, MA USA 02451); USA, Cat. Nr. MA5-15738).

### 4.5. Behavioral Testing

All the behavioral experiments described here were conducted at morning hours, throughout the light phase of the imposed light/dark cycle, in a noise-isolated room. Before the start of the experiments, all the animals went through a period of daily handling for 5 min, conducted by the experimenter and implemented to familiarize the animals with the experimenter and the handling, thus reducing stress of the animals derived from the manipulations required for the experiments. On the testing days and before the beginning of the experiments, the animals were allowed to rest for 1 h in their home cages so that they could become habituated to the testing room.

### 4.6. Open Field Test

The Open Field test (OFT) is commonly used in experiments used with rodents in order to monitor basic locomotor activity as well as some aspects of anxiety-related behaviors [174]. Experiments in the OFT were conducted following protocols previously described by our group [65,66,67,175,176]. In brief, mice were placed in the center of an open field arena, illuminated at ~300 lux, consisting of a four white-mate plastic-walled box (30 × 30 × 30 cm). Animals were allowed to freely occupy the arena for 10 min, and were then returned to their home cages. The OFT box was then thoroughly cleaned using 70% ethanol and allowed to dry for several minutes before reuse. In order to examine the animals’ behavior in the open field, the cumulative time that animals spent in the center of the arena as well as the total distance traveled were considered (Figure 9; see also [177]). All the OFT routines were digitalized using a zenithal high-definition digital video camera. Spatial/Temporal behavioral factors were evaluated using the XT12 version of the Ethovision software package (Noldus, Wageningen, The Netherlands).

### 4.7. Elevated Plus Maze

Behavioral measurements using the Elevated Plus Maze (EPM) (see Figure 9) were conducted as previously described [65,178]. This test continues to be widely used for the examination of anxiety-like behaviors in experimental animal models, including mice and rats, and relies on the instinctive fear response that animals display to open, elevated zones. In the case of mice and rats with healthy/natural self-preservation instincts, they will initially have the generalized tendence to avoid entering to open/elevated potentially dangerous areas, and would remain in or move towards enclosed zone in search for safety [179]. The experiments were conducted using a customized plus-shaped platform made of white-mate Plexiglas (elevated 50 cm above ground) having two of its opposing arms enclosed by 40 cm high walls. Time in the open arms as well as open arm latency to first were examined in the EPM. All arms had 50 cm in length and were 10 cm wide. The open arms were exposed to an illumination of ~110 lux, whereas ~15 lux illuminated the enclosed arms. The testing sessions (5 min each) begin immediately after having placed the animal at the central square area, with animals positioned facing towards one of the open arms, and animals were allowed to freely move across the maze settings. The EPM behavioral performance was digitalized using a zenithal high-definition digital video camera. Spatial/Temporal behavioral factors were evaluated using the XT12 version of the Ethovision software package (Noldus, Wageningen, The Netherlands). Behavioral parameters in the EPM were examined following protocols previously described [180].

### 4.8. Statistical Analysis

All data analyses were conducted using the GraphPad-Prism-9 software package (version 9.5.1(733), GraphPad Software, 225 Franklin Street. Fl. 26, Boston, MA 02110, USA). In order to comply with the 3Rs regulation and reduce as much as possible the number of animals, for the electrophysiological and behavioral analyses, statistical calculations were based on power analysis to estimate the minimum sample size required, using the G*Power software (version 3.1.9.7). For biochemical studies, since the normalized data obtained from examinations using western blot are relative/semi-quantitative, we used a small number of animals in agreement with related published studies by other groups [181]. Unpaired two-tailed *t*-tests (with confidence level set to 95%) was used to examine differences of means between groups in behavioral experiments. Two-way ANOVA (Alpha 0.05) with Tukey’s multiple comparisons test was used to examine data from WB experiments. Three- and two-way repeated measures (RM)-ANOVA with Tukey multiple comparisons, and/or Bonferroni´s and Geisser–Greenhouse´s corrections (and alpha set to 0.05), were used for the data derived from the electrophysiological analyses as in each case indicated in the main text. For bias control, data were examined by researchers who were blind to the experimental groups.

## Data Availability

All the data derived from this work are included in the article.

## Abbreviations

aCSF = artificial cerebrospinal fluid; EMP = elevated plus maze; fEPSPs = field excitatory postsynaptic potentials; HFS = high frequency stimulation; HPA = hypothalamic–pituitary–adrenal; KO = knockout; LTP = long-term potentiation; PTP = post-tetanic potentiation; I/O = input/output; miRNA = microRNA; OFT = open field test; WB = western blot; WT = wild type.

## References

[1] Thau L., Gandhi J., Sharma S. (2023). Physiology, Cortisol. StatPearls.

[2] Oakley R.H., Cidlowski J.A. (2013). The biology of the glucocorticoid receptor: New signaling mechanisms in health and disease. J. Allergy Clin. Immunol..

[3] Payne J.D., Jackson E.D., Hoscheidt S., Ryan L., Jacobs W.J., Nadel L. (2007). Stress administered prior to encoding impairs neutral but enhances emotional long-term episodic memories. Learn. Mem. (Cold Spring Harb. N. Y.).

[4] Roozendaal B., Okuda S., Van der Zee E.A., McGaugh J.L. (2006). Glucocorticoid enhancement of memory requires arousal-induced noradrenergic activation in the basolateral amygdala. Proc. Natl. Acad. Sci. USA.

[5] Lupien S.J., Maheu F., Tu M., Fiocco A., Schramek T.E. (2007). The effects of stress and stress hormones on human cognition: Implications for the field of brain and cognition. Brain Cogn..

[6] Hayley S., Poulter M.O., Merali Z., Anisman H. (2005). The pathogenesis of clinical depression: Stressor- and cytokine-induced alterations of neuroplasticity. Neuroscience.

[7] Pariante C.M., Lightman S.L. (2008). The HPA axis in major depression: Classical theories and new developments. Trends Neurosci..

[8] Silverman M.N., Sternberg E.M. (2012). Glucocorticoid regulation of inflammation and its functional correlates: From HPA axis to glucocorticoid receptor dysfunction. Ann. N. Y. Acad. Sci..

[9] Munck A., Guyre P.M., Holbrook N.J. (1984). Physiological functions of glucocorticoids in stress and their relation to pharmacological actions. Endocr. Rev..

[10] Aguilera G. (1994). Regulation of pituitary ACTH secretion during chronic stress. Front. Neuroendocr..

[11] Varghese F.P., Brown E.S. (2001). The Hypothalamic-Pituitary-Adrenal Axis in Major Depressive Disorder: A Brief Primer for Primary Care Physicians. Prim. Care Companion J. Clin. Psychiatry.

[12] Owens M.J., Nemeroff C.B. (1993). The role of corticotropin-releasing factor in the pathophysiology of affective and anxiety disorders: Laboratory and clinical studies. Ciba Found. Symp..

[13] Young E.A., Haskett R.F., Murphy-Weinberg V., Watson S.J., Akil H. (1991). Loss of glucocorticoid fast feedback in depression. Arch. Gen. Psychiatry.

[14] Lipov E., Kelzenberg B., Rothfeld C., Abdi S. (2012). Modulation of NGF by cortisol and the Stellate Ganglion Block—Is this the missing link between memory consolidation and PTSD?. Med. Hypotheses.

[15] Sagmeister M.S., Harper L., Hardy R.S. (2022). Cortisol excess in chronic kidney disease—A review of changes and impact on mortality. Front. Endocrinol..

[16] Brown E.S., Rush A.J., McEwen B.S. (1999). Hippocampal remodeling and damage by corticosteroids: Implications for mood disorders. Neuropsychopharmacology.

[17] Lupien S.J., McEwen B.S. (1997). The acute effects of corticosteroids on cognition: Integration of animal and human model studies. Brain Res..

[18] Charmandari E., Tsigos C., Chrousos G. (2005). Endocrinology of the stress response. Annu. Rev. Physiol..

[19] Sapolsky R.M., Romero L.M., Munck A.U. (2000). How do glucocorticoids influence stress responses? Integrating permissive, suppressive, stimulatory, and preparative actions. Endocr. Rev..

[20] Brinks V., van der Mark M., de Kloet R., Oitzl M. (2007). Emotion and cognition in high and low stress sensitive mouse strains: A combined neuroendocrine and behavioral study in BALB/c and C57BL/6J mice. Front. Behav. Neurosci..

[21] Eadie B.D., Zhang W.N., Boehme F., Gil-Mohapel J., Kainer L., Simpson J.M., Christie B.R. (2009). Fmr1 knockout mice show reduced anxiety and alterations in neurogenesis that are specific to the ventral dentate gyrus. Neurobiol. Dis..

[22] Zhao S., Xu X., Xie G., Zhang T. (2022). Chronic corticosterone exposure impairs emotional regulation and cognitive function through disturbing neural oscillations in mice. Behav. Brain Res..

[23] Scoville W.B., Milner B. (1957). Loss of recent memory after bilateral hippocampal lesions. J. Neurol. Neurosurg. Psychiatry.

[24] Scoville W.B., Milner B. (2000). Loss of recent memory after bilateral hippocampal lesions. J. Neuropsychiatry Clin. Neurosci..

[25] Milner B. (2005). The medial temporal-lobe amnesic syndrome. Psychiatr. Clin. N. Am..

[26] Milner B., Klein D. (2016). Loss of recent memory after bilateral hippocampal lesions: Memory and memories-looking back and looking forward. J. Neurol. Neurosurg. Psychiatry.

[27] Izquierdo I., Furini C.R., Myskiw J.C. (2016). Fear Memory. Physiol. Rev..

[28] Patel P.D., Katz M., Karssen A.M., Lyons D.M. (2008). Stress-induced changes in corticosteroid receptor expression in primate hippocampus and prefrontal cortex. Psychoneuroendocrinology.

[29] Sapolsky R.M., Krey L.C., McEwen B.S. (1984). Glucocorticoid-sensitive hippocampal neurons are involved in terminating the adrenocortical stress response. Proc. Natl. Acad. Sci. USA.

[30] Dahmen B., Puetz V.B., Scharke W., von Polier G.G., Herpertz-Dahlmann B., Konrad K. (2018). Effects of Early-Life Adversity on Hippocampal Structures and Associated HPA Axis Functions. Dev. Neurosci..

[31] Dai S., Mo Y., Wang Y., Xiang B., Liao Q., Zhou M., Li X., Li Y., Xiong W., Li G. (2020). Chronic Stress Promotes Cancer Development. Front. Oncol..

[32] Clow A., Thorn L., Evans P., Hucklebridge F. (2004). The awakening cortisol response: Methodological issues and significance. Stress.

[33] Frodl T., O’Keane V. (2013). How does the brain deal with cumulative stress? A review with focus on developmental stress, HPA axis function and hippocampal structure in humans. Neurobiol. Dis..

[34] Alfarez D.N., Wiegert O., Joels M., Krugers H.J. (2002). Corticosterone and stress reduce synaptic potentiation in mouse hippocampal slices with mild stimulation. Neuroscience.

[35] Rey M., Carlier E., Talmi M., Soumireu-Mourat B. (1994). Corticosterone effects on long-term potentiation in mouse hippocampal slices. Neuroendocrinology.

[36] Abraham W.C., Bear M.F. (1996). Metaplasticity: The plasticity of synaptic plasticity. Trends Neurosci..

[37] Caliskan G., Stork O. (2018). Hippocampal network oscillations as mediators of behavioural metaplasticity: Insights from emotional learning. Neurobiol. Learn. Mem..

[38] Holland L.L., Wagner J.J. (1998). Primed facilitation of homosynaptic long-term depression and depotentiation in rat hippocampus. J. Neurosci..

[39] Ambros V. (2001). microRNAs: Tiny regulators with great potential. Cell..

[40] Bartel D.P. (2004). MicroRNAs: Genomics, biogenesis, mechanism, and function. Cell..

[41] Edbauer D., Neilson J.R., Foster K.A., Wang C.F., Seeburg D.P., Batterton M.N., Tada T., Dolan B.M., Sharp P.A., Sheng M. (2010). Regulation of synaptic structure and function by FMRP-associated microRNAs miR-125b and miR-132. Neuron.

[42] Oliver R.J., Mandyam C.D. (2018). Regulation of Adult Neurogenesis by Non-coding RNAs: Implications for Substance Use Disorders. Front. Neurosci..

[43] Stojanovic T., Benes H., Awad A., Bormann D., Monje F.J. (2020). Nicotine abolishes memory-related synaptic strengthening and promotes synaptic depression in the neurogenic dentate gyrus of miR-132/212 knockout mice. Addict. Biol..

[44] Baby N., Alagappan N., Dheen S.T., Sajikumar S. (2020). MicroRNA-134-5p inhibition rescues long-term plasticity and synaptic tagging/capture in an Abeta(1-42)-induced model of Alzheimer’s disease. Aging Cell..

[45] Berentsen B., Patil S., Ronnestad K., Goff K.M., Pajak M., Simpson T.I., Wibrand K., Bramham C.R. (2020). MicroRNA-34a Acutely Regulates Synaptic Efficacy in the Adult Dentate Gyrus In Vivo. Mol. Neurobiol..

[46] Zhang H.P., Liu X.L., Chen J.J., Cheng K., Bai S.J., Zheng P., Zhou C.J., Wang W., Wang H.Y., Zhong L.M. (2020). Circulating microRNA 134 sheds light on the diagnosis of major depressive disorder. Transl. Psychiatry.

[47] Liu D.Y., Zhang L. (2019). MicroRNA-132 promotes neurons cell apoptosis and activates Tau phosphorylation by targeting GTDC-1 in Alzheimer’s disease. Eur. Rev. Med. Pharm. Sci..

[48] Kumar S., Reddy P.H. (2019). A New Discovery of MicroRNA-455-3p in Alzheimer’s Disease. J. Alzheimers Dis..

[49] Herrera-Espejo S., Santos-Zorrozua B., Alvarez-Gonzalez P., Lopez-Lopez E., Garcia-Orad A. (2019). A Systematic Review of MicroRNA Expression as Biomarker of Late-Onset Alzheimer’s Disease. Mol. Neurobiol..

[50] Salta E., De Strooper B. (2017). microRNA-132: A key noncoding RNA operating in the cellular phase of Alzheimer’s disease. FASEB J..

[51] Hernandez-Rapp J., Rainone S., Goupil C., Dorval V., Smith P.Y., Saint-Pierre M., Vallee M., Planel E., Droit A., Calon F. (2016). microRNA-132/212 deficiency enhances Abeta production and senile plaque deposition in Alzheimer’s disease triple transgenic mice. Sci. Rep..

[52] Salta E., Sierksma A., Vanden Eynden E., De Strooper B. (2016). miR-132 loss de-represses ITPKB and aggravates amyloid and TAU pathology in Alzheimer’s brain. EMBO Mol. Med..

[53] Aten S., Hansen K.F., Hoyt K.R., Obrietan K. (2016). The miR-132/212 locus: A complex regulator of neuronal plasticity, gene expression and cognition. RNA Dis..

[54] Hansen K.F., Sakamoto K., Aten S., Snider K.H., Loeser J., Hesse A.M., Page C.E., Pelz C., Arthur J.S., Impey S. (2016). Targeted deletion of miR-132/-212 impairs memory and alters the hippocampal transcriptome. Learn. Mem..

[55] Mendoza-Viveros L., Chiang C.K., Ong J.L.K., Hegazi S., Cheng A.H., Bouchard-Cannon P., Fana M., Lowden C., Zhang P., Bothorel B. (2017). miR-132/212 Modulates Seasonal Adaptation and Dendritic Morphology of the Central Circadian Clock. Cell. Rep..

[56] Remenyi J., Hunter C.J., Cole C., Ando H., Impey S., Monk C.E., Martin K.J., Barton G.J., Hutvagner G., Arthur J.S. (2010). Regulation of the miR-212/132 locus by MSK1 and CREB in response to neurotrophins. Biochem. J..

[57] Remenyi J., van den Bosch M.W., Palygin O., Mistry R.B., McKenzie C., Macdonald A., Hutvagner G., Arthur J.S., Frenguelli B.G., Pankratov Y. (2013). miR-132/212 knockout mice reveal roles for these miRNAs in regulating cortical synaptic transmission and plasticity. PLoS ONE.

[58] Wanet A., Tacheny A., Arnould T., Renard P. (2012). miR-212/132 expression and functions: Within and beyond the neuronal compartment. Nucleic Acids Res..

[59] Tognini P., Pizzorusso T. (2012). MicroRNA212/132 family: Molecular transducer of neuronal function and plasticity. Int. J. Biochem. Cell. Biol..

[60] Bormann D., Stojanovic T., Cicvaric A., Schuld G.J., Cabatic M., Ankersmit H.J., Monje F.J. (2021). miRNA-132/212 Gene-Deletion Aggravates the Effect of Oxygen-Glucose Deprivation on Synaptic Functions in the Female Mouse Hippocampus. Cells.

[61] Stojanovic T., Velarde Gamez D., Schuld G.J., Bormann D., Cabatic M., Uhrin P., Lubec G., Monje F.J. (2022). Age-Dependent and Pathway-Specific Bimodal Action of Nicotine on Synaptic Plasticity in the Hippocampus of Mice Lacking the miR-132/212 Genes. Cells.

[62] Ronovsky M., Zambon A., Cicvaric A., Boehm V., Hoesel B., Moser B.A., Yang J., Schmid J.A., Haubensak W.E., Monje F.J. (2019). A role for miR-132 in learned safety. Sci. Rep..

[63] Pu Z., Krugers H.J., Joels M. (2009). Beta-adrenergic facilitation of synaptic plasticity in the rat basolateral amygdala in vitro is gradually reversed by corticosterone. Learn. Mem. (Cold Spring Harb. N. Y.).

[64] Kouhnavardi S., Ecevitoglu A., Dragacevic V., Sanna F., Arias-Sandoval E., Kalaba P., Kirchhofer M., Lubec J., Niello M., Holy M. (2022). A Novel and Selective Dopamine Transporter Inhibitor, (S)-MK-26, Promotes Hippocampal Synaptic Plasticity and Restores Effort-Related Motivational Dysfunctions. Biomolecules.

[65] Cicvaric A., Sachernegg H.M., Stojanovic T., Symmank D., Smani T., Moeslinger T., Uhrin P., Monje F.J. (2019). Podoplanin Gene Disruption in Mice Promotes in vivo Neural Progenitor Cells Proliferation, Selectively Impairs Dentate Gyrus Synaptic Depression and Induces Anxiety-Like Behaviors. Front. Cell. Neurosci..

[66] Cicvaric A., Yang J., Bulat T., Zambon A., Dominguez-Rodriguez M., Kuhn R., Sadowicz M.G., Siwert A., Egea J., Pollak D.D. (2018). Enhanced synaptic plasticity and spatial memory in female but not male FLRT2-haplodeficient mice. Sci. Rep..

[67] Cicvaric A., Bulat T., Bormann D., Yang J., Auer B., Milenkovic I., Cabatic M., Milicevic R., Monje F.J. (2018). Sustained consumption of cocoa-based dark chocolate enhances seizure-like events in the mouse hippocampus. Food Funct..

[68] Cicvaric A., Yang J., Krieger S., Khan D., Kim E.J., Dominguez-Rodriguez M., Cabatic M., Molz B., Acevedo Aguilar J.P., Milicevic R. (2016). The brain-tumor related protein podoplanin regulates synaptic plasticity and hippocampus-dependent learning and memory. Ann. Med..

[69] Kim E.J., Monje F.J., Li L., Hoger H., Pollak D.D., Lubec G. (2013). Alzheimer’s disease risk factor lymphocyte-specific protein tyrosine kinase regulates long-term synaptic strengthening, spatial learning and memory. Cell. Mol. Life Sci..

[70] Monje F.J., Kim E.J., Pollak D.D., Cabatic M., Li L., Baston A., Lubec G. (2012). Focal adhesion kinase regulates neuronal growth, synaptic plasticity and hippocampus-dependent spatial learning and memory. Neuro-Signals.

[71] Maggio N., Segal M. (2007). Striking variations in corticosteroid modulation of long-term potentiation along the septotemporal axis of the hippocampus. J. Neurosci..

[72] Maggio N., Segal M. (2007). Unique regulation of long term potentiation in the rat ventral hippocampus. Hippocampus.

[73] Nagy V., Hollstein R., Pai T.P., Herde M.K., Buphamalai P., Moeseneder P., Lenartowicz E., Kavirayani A., Korenke G.C., Kozieradzki I. (2019). HACE1 deficiency leads to structural and functional neurodevelopmental defects. Neurol. Genet..

[74] Fell C.W., Hagelkruys A., Cicvaric A., Horrer M., Liu L., Li J.S.S., Stadlmann J., Polyansky A.A., Mereiter S., Tejada M.A. (2022). FIBCD1 is an endocytic GAG receptor associated with a novel neurodevelopmental disorder. EMBO Mol. Med..

[75] Nicoll R.A., Malenka R.C. (1999). Expression mechanisms underlying NMDA receptor-dependent long-term potentiation. Ann. N. Y. Acad. Sci..

[76] Schulz P.E., Cook E.P., Johnston D. (1994). Changes in paired-pulse facilitation suggest presynaptic involvement in long-term potentiation. J. Neurosci..

[77] Bortolotto Z.A., Collingridge G.L. (2000). A role for protein kinase C in a form of metaplasticity that regulates the induction of long-term potentiation at CA1 synapses of the adult rat hippocampus. Eur. J. Neurosci..

[78] Nestler E.J. (2002). Common molecular and cellular substrates of addiction and memory. Neurobiol. Learn. Mem..

[79] Hansen K.F., Karelina K., Sakamoto K., Wayman G.A., Impey S., Obrietan K. (2013). miRNA-132: A dynamic regulator of cognitive capacity. Brain Struct. Funct..

[80] Fisher M.L., LeMalefant R.M., Zhou L., Huang G., Turner J.R. (2017). Distinct Roles of CREB Within the Ventral and Dorsal Hippocampus in Mediating Nicotine Withdrawal Phenotypes. Neuropsychopharmacology.

[81] Barco A., Patterson S., Alarcon J.M., Gromova P., Mata-Roig M., Morozov A., Kandel E.R. (2005). Gene expression profiling of facilitated L-LTP in VP16-CREB mice reveals that BDNF is critical for the maintenance of LTP and its synaptic capture. Neuron.

[82] Magill S.T., Cambronne X.A., Luikart B.W., Lioy D.T., Leighton B.H., Westbrook G.L., Mandel G., Goodman R.H. (2010). microRNA-132 regulates dendritic growth and arborization of newborn neurons in the adult hippocampus. Proc. Natl. Acad. Sci. USA.

[83] Haghparast A., Taslimi Z., Ramin M., Azizi P., Khodagholi F., Hassanpour-Ezatti M. (2011). Changes in phosphorylation of CREB, ERK, and c-fos induction in rat ventral tegmental area, hippocampus and prefrontal cortex after conditioned place preference induced by chemical stimulation of lateral hypothalamus. Behav. Brain Res..

[84] Hadar A., Milanesi E., Walczak M., Puzianowska-Kuznicka M., Kuznicki J., Squassina A., Niola P., Chillotti C., Attems J., Gozes I. (2018). SIRT1, miR-132 and miR-212 link human longevity to Alzheimer’s Disease. Sci. Rep..

[85] Herskovits A.Z., Guarente L. (2014). SIRT1 in neurodevelopment and brain senescence. Neuron.

[86] Luikart B.W., Bensen A.L., Washburn E.K., Perederiy J.V., Su K.G., Li Y., Kernie S.G., Parada L.F., Westbrook G.L. (2011). miR-132 mediates the integration of newborn neurons into the adult dentate gyrus. PLoS ONE.

[87] Jiang Y., Botchway B.O.A., Hu Z., Fang M. (2019). Overexpression of SIRT1 Inhibits Corticosterone-Induced Autophagy. Neuroscience.

[88] Aten S., Page C.E., Kalidindi A., Wheaton K.L., Niraula A., Godbout J.P., Hoyt K.R., Obrietan K. (2018). Data highlighting the expression of two miR-132/212 target genes-Sirt1 and Pten-after chronic stress. Data Brief..

[89] Arthur J.S. (2008). MSK activation and physiological roles. Front. Biosci..

[90] Hauge C., Frodin M. (2006). RSK and MSK in MAP kinase signalling. J. Cell. Sci..

[91] Chandramohan Y., Droste S.K., Arthur J.S., Reul J.M. (2008). The forced swimming-induced behavioural immobility response involves histone H3 phospho-acetylation and c-Fos induction in dentate gyrus granule neurons via activation of the N-methyl-D-aspartate/extracellular signal-regulated kinase/mitogen- and stress-activated kinase signalling pathway. Eur. J. Neurosci..

[92] Chwang W.B., Arthur J.S., Schumacher A., Sweatt J.D. (2007). The nuclear kinase mitogen- and stress-activated protein kinase 1 regulates hippocampal chromatin remodeling in memory formation. J. Neurosci..

[93] Gutierrez-Mecinas M., Trollope A.F., Collins A., Morfett H., Hesketh S.A., Kersante F., Reul J.M. (2011). Long-lasting behavioral responses to stress involve a direct interaction of glucocorticoid receptors with ERK1/2-MSK1-Elk-1 signaling. Proc. Natl. Acad. Sci. USA.

[94] Sanchez-Puelles C., Calleja-Felipe M., Ouro A., Bougamra G., Arroyo A., Diez I., Erramuzpe A., Cortes J., Martinez-Hernandez J., Lujan R. (2020). PTEN Activity Defines an Axis for Plasticity at Cortico-Amygdala Synapses and Influences Social Behavior. Cereb. Cortex.

[95] Wang P., Mei F., Hu J., Zhu M., Qi H., Chen X., Li R., McNutt M.A., Yin Y. (2017). PTENalpha Modulates CaMKII Signaling and Controls Contextual Fear Memory and Spatial Learning. Cell. Rep..

[96] Sperow M., Berry R.B., Bayazitov I.T., Zhu G., Baker S.J., Zakharenko S.S. (2012). Phosphatase and tensin homologue (PTEN) regulates synaptic plasticity independently of its effect on neuronal morphology and migration. J. Physiol..

[97] Silva A.R., Santos A.C., Farfel J.M., Grinberg L.T., Ferretti R.E., Campos A.H., Cunha I.W., Begnami M.D., Rocha R.M., Carraro D.M. (2014). Repair of oxidative DNA damage, cell-cycle regulation and neuronal death may influence the clinical manifestation of Alzheimer’s disease. PLoS ONE.

[98] Quan Q., Qian Y., Li X., Li M. (2019). CDK5 Participates in Amyloid-beta Production by Regulating PPARgamma Phosphorylation in Primary Rat Hippocampal Neurons. J. Alzheimers Dis..

[99] Liu W., Zhou Y., Liang R., Zhang Y. (2019). Inhibition of cyclin-dependent kinase 5 activity alleviates diabetes-related cognitive deficits. FASEB J..

[100] Brossaud J., Roumes H., Helbling J.C., Moisan M.P., Pallet V., Ferreira G., Biyong E.F., Redonnet A., Corcuff J.B. (2017). Retinoic acid increases glucocorticoid receptor phosphorylation via cyclin-dependent kinase 5. Mol. Cell. Neurosci..

[101] Jin X., Sasamoto K., Nagai J., Yamazaki Y., Saito K., Goshima Y., Inoue T., Ohshima T. (2016). Phosphorylation of CRMP2 by Cdk5 Regulates Dendritic Spine Development of Cortical Neuron in the Mouse Hippocampus. Neural Plast..

[102] Tomizawa K., Ohta J., Matsushita M., Moriwaki A., Li S.T., Takei K., Matsui H. (2002). Cdk5/p35 regulates neurotransmitter release through phosphorylation and downregulation of P/Q-type voltage-dependent calcium channel activity. J. Neurosci..

[103] Iijima K., Ando K., Takeda S., Satoh Y., Seki T., Itohara S., Greengard P., Kirino Y., Nairn A.C., Suzuki T. (2000). Neuron-specific phosphorylation of Alzheimer’s beta-amyloid precursor protein by cyclin-dependent kinase 5. J. Neurochem..

[104] Agasse F., Mendez-David I., Christaller W., Carpentier R., Braz B.Y., David D.J., Saudou F., Humbert S. (2020). Chronic Corticosterone Elevation Suppresses Adult Hippocampal Neurogenesis by Hyperphosphorylating Huntingtin. Cell. Rep..

[105] Mitic M., Simic I., Djordjevic J., Radojcic M.B., Adzic M. (2013). Gender-specific effects of fluoxetine on hippocampal glucocorticoid receptor phosphorylation and behavior in chronically stressed rats. Neuropharmacology.

[106] Adzic M., Djordjevic J., Djordjevic A., Niciforovic A., Demonacos C., Radojcic M., Krstic-Demonacos M. (2009). Acute or chronic stress induce cell compartment-specific phosphorylation of glucocorticoid receptor and alter its transcriptional activity in Wistar rat brain. J. Endocrinol..

[107] Shen Y., Chen L., Zhang Y., Du J., Hu J., Bao H., Xing Y., Si Y. (2021). Phosphatase and Tensin Homolog Deleted on Chromosome Ten Knockdown Attenuates Cognitive Deficits by Inhibiting Neuroinflammation in a Mouse Model of Perioperative Neurocognitive Disorder. Neuroscience.

[108] Choi G.E., Lee H.J., Chae C.W., Cho J.H., Jung Y.H., Kim J.S., Kim S.Y., Lim J.R., Han H.J. (2021). BNIP3L/NIX-mediated mitophagy protects against glucocorticoid-induced synapse defects. Nat. Commun..

[109] Chen D., Lan G., Li R., Mei Y., Shui X., Gu X., Wang L., Zhang T., Gan C.L., Xia Y. (2022). Melatonin ameliorates tau-related pathology via the miR-504-3p and CDK5 axis in Alzheimer’s disease. Transl. Neurodegener..

[110] Inouye M.O., Colameo D., Ammann I., Winterer J., Schratt G. (2022). miR-329- and miR-495-mediated Prr7 down-regulation is required for homeostatic synaptic depression in rat hippocampal neurons. Life Sci. Alliance.

[111] Li Y., Fan C., Wang L., Lan T., Gao R., Wang W., Yu S.Y. (2021). MicroRNA-26a-3p rescues depression-like behaviors in male rats via preventing hippocampal neuronal anomalies. J. Clin. Investig..

[112] Aten S., Page C.E., Kalidindi A., Wheaton K., Niraula A., Godbout J.P., Hoyt K.R., Obrietan K. (2019). miR-132/212 is induced by stress and its dysregulation triggers anxiety-related behavior. Neuropharmacology.

[113] Travis S.G., Coupland N.J., Hegadoren K., Silverstone P.H., Huang Y., Carter R., Fujiwara E., Seres P., Malykhin N.V. (2016). Effects of cortisol on hippocampal subfields volumes and memory performance in healthy control subjects and patients with major depressive disorder. J. Affect. Disord..

[114] Keller J., Gomez R., Williams G., Lembke A., Lazzeroni L., Murphy G.M., Schatzberg A.F. (2017). HPA axis in major depression: Cortisol, clinical symptomatology and genetic variation predict cognition. Mol. Psychiatry.

[115] Dominguez-Borras J., Vuilleumier P. (2022). Amygdala function in emotion, cognition, and behavior. Handb. Clin. Neurol..

[116] Simic G., Tkalcic M., Vukic V., Mulc D., Spanic E., Sagud M., Olucha-Bordonau F.E., Vuksic M., P R.H. (2021). Understanding Emotions: Origins and Roles of the Amygdala. Biomolecules.

[117] Kim J.J., Lee H.J., Han J.S., Packard M.G. (2001). Amygdala is critical for stress-induced modulation of hippocampal long-term potentiation and learning. J. Neurosci..

[118] Segall L.A., Milet A., Tronche F., Amir S. (2009). Brain glucocorticoid receptors are necessary for the rhythmic expression of the clock protein, PERIOD2, in the central extended amygdala in mice. Neurosci. Lett..

[119] Li Y., He Y., Fan H., Wang Z., Huang J., Wen G., Wang X., Xie Q., Qiu P. (2021). Brain-derived neurotrophic factor upregulates synaptic GluA1 in the amygdala to promote depression in response to psychological stress. Biochem. Pharm..

[120] Mackiewicz K.L., Sarinopoulos I., Cleven K.L., Nitschke J.B. (2006). The effect of anticipation and the specificity of sex differences for amygdala and hippocampus function in emotional memory. Proc. Natl. Acad. Sci. USA.

[121] Fanselow M.S., Dong H.W. (2010). Are the dorsal and ventral hippocampus functionally distinct structures?. Neuron.

[122] Floriou-Servou A., von Ziegler L., Stalder L., Sturman O., Privitera M., Rassi A., Cremonesi A., Thony B., Bohacek J. (2018). Distinct Proteomic, Transcriptomic, and Epigenetic Stress Responses in Dorsal and Ventral Hippocampus. Biol. Psychiatry.

[123] Ohara S., Sato S., Tsutsui K., Witter M.P., Iijima T. (2013). Organization of multisynaptic inputs to the dorsal and ventral dentate gyrus: Retrograde trans-synaptic tracing with rabies virus vector in the rat. PLoS ONE.

[124] Steullet P., Cabungcal J.H., Kulak A., Kraftsik R., Chen Y., Dalton T.P., Cuenod M., Do K.Q. (2010). Redox dysregulation affects the ventral but not dorsal hippocampus: Impairment of parvalbumin neurons, gamma oscillations, and related behaviors. J. Neurosci..

[125] Igarashi K.M., Ito H.T., Moser E.I., Moser M.B. (2014). Functional diversity along the transverse axis of hippocampal area CA1. FEBS Lett..

[126] Moser M.B., Moser E.I. (1998). Functional differentiation in the hippocampus. Hippocampus.

[127] Strange B.A., Witter M.P., Lein E.S., Moser E.I. (2014). Functional organization of the hippocampal longitudinal axis. Nat. Rev..

[128] Torrisi S.A., Lavanco G., Maurel O.M., Gulisano W., Laudani S., Geraci F., Grasso M., Barbagallo C., Caraci F., Bucolo C. (2021). A novel arousal-based individual screening reveals susceptibility and resilience to PTSD-like phenotypes in mice. Neurobiol. Stress..

[129] Maurel O.M., Torrisi S.A., Barbagallo C., Purrello M., Salomone S., Drago F., Ragusa M., Leggio G.M. (2021). Dysregulation of miR-15a-5p, miR-497a-5p and miR-511-5p Is Associated with Modulation of BDNF and FKBP5 in Brain Areas of PTSD-Related Susceptible and Resilient Mice. Int. J. Mol. Sci..

[130] Belovicova K., Bogi E., Csatlosova K., Dubovicky M. (2017). Animal tests for anxiety-like and depression-like behavior in rats. Interdiscip. Toxicol..

[131] Hogg S. (1996). A review of the validity and variability of the elevated plus-maze as an animal model of anxiety. Pharmacol. Biochem. Behav..

[132] Bourin M., Hascoet M. (2003). The mouse light/dark box test. Eur. J. Pharmacol..

[133] Hagenbuch N., Feldon J., Yee B.K. (2006). Use of the elevated plus-maze test with opaque or transparent walls in the detection of mouse strain differences and the anxiolytic effects of diazepam. Behav. Pharmacol..

[134] Ramos A. (2008). Animal models of anxiety: Do I need multiple tests?. Trends Pharmacol. Sci..

[135] Violle N., Balandras F., Le Roux Y., Desor D., Schroeder H. (2009). Variations in illumination, closed wall transparency and/or extramaze space influence both baseline anxiety and response to diazepam in the rat elevated plus-maze. Behav. Brain Res..

[136] Miller S.M., Piasecki C.C., Lonstein J.S. (2011). Use of the light-dark box to compare the anxiety-related behavior of virgin and postpartum female rats. Pharmacol. Biochem. Behav..

[137] Steimer T. (2011). Animal models of anxiety disorders in rats and mice: Some conceptual issues. Dialogues Clin. Neurosci..

[138] Mineur Y.S., Fote G.M., Blakeman S., Cahuzac E.L., Newbold S.A., Picciotto M.R. (2016). Multiple Nicotinic Acetylcholine Receptor Subtypes in the Mouse Amygdala Regulate Affective Behaviors and Response to Social Stress. Neuropsychopharmacology.

[139] Pidoplichko V.I., Prager E.M., Aroniadou-Anderjaska V., Braga M.F. (2013). alpha7-Containing nicotinic acetylcholine receptors on interneurons of the basolateral amygdala and their role in the regulation of the network excitability. J. Neurophysiol..

[140] Laviolette S.R. (2021). Molecular and neuronal mechanisms underlying the effects of adolescent nicotine exposure on anxiety and mood disorders. Neuropharmacology.

[141] Zhang M., Bian Z. (2021). Alzheimer’s Disease and microRNA-132: A Widespread Pathological Factor and Potential Therapeutic Target. Front. Neurosci..

[142] Alkadhi K.A., Alzoubi K.H., Srivareerat M., Tran T.T. (2011). Chronic psychosocial stress exacerbates impairment of synaptic plasticity in beta-amyloid rat model of Alzheimer’s disease: Prevention by nicotine. Curr. Alzheimer Res..

[143] Alkadhi K.A., Srivareerat M., Tran T.T. (2010). Intensification of long-term memory deficit by chronic stress and prevention by nicotine in a rat model of Alzheimer’s disease. Mol. Cell. Neurosci..

[144] Musazzi L., Tornese P., Sala N., Lee F.S., Popoli M., Ieraci A. (2022). Acute stress induces an aberrant increase of presynaptic release of glutamate and cellular activation in the hippocampus of BDNF(Val/Met) mice. J. Cell. Physiol..

[145] Tan Y., Rouse J., Zhang A., Cariati S., Cohen P., Comb M.J. (1996). FGF and stress regulate CREB and ATF-1 via a pathway involving p38 MAP kinase and MAPKAP kinase-2. EMBO J..

[146] Hernandez-Rapp J., Smith P.Y., Filali M., Goupil C., Planel E., Magill S.T., Goodman R.H., Hebert S.S. (2015). Memory formation and retention are affected in adult miR-132/212 knockout mice. Behav. Brain Res..

[147] Lee Y.J., Kim H.R., Lee C.Y., Hyun S.A., Ko M.Y., Lee B.S., Hwang D.Y., Ka M. (2020). 2-Phenylethylamine (PEA) Ameliorates Corticosterone-Induced Depression-Like Phenotype via the BDNF/TrkB/CREB Signaling Pathway. Int. J. Mol. Sci..

[148] Lu Y., Sareddy G.R., Wang J., Wang R., Li Y., Dong Y., Zhang Q., Liu J., O’Connor J.C., Xu J. (2019). Neuron-Derived Estrogen Regulates Synaptic Plasticity and Memory. J. Neurosci..

[149] Okun E., Griffioen K., Barak B., Roberts N.J., Castro K., Pita M.A., Cheng A., Mughal M.R., Wan R., Ashery U. (2010). Toll-like receptor 3 inhibits memory retention and constrains adult hippocampal neurogenesis. Proc. Natl. Acad. Sci. USA.

[150] Hansen K.F., Sakamoto K., Wayman G.A., Impey S., Obrietan K. (2010). Transgenic miR132 alters neuronal spine density and impairs novel object recognition memory. PLoS ONE.

[151] Lambert T.J., Storm D.R., Sullivan J.M. (2010). MicroRNA132 modulates short-term synaptic plasticity but not basal release probability in hippocampal neurons. PLoS ONE.

[152] Silva A.J., Kogan J.H., Frankland P.W., Kida S. (1998). CREB and memory. Annu. Rev. Neurosci..

[153] Meyer H.C., Odriozola P., Cohodes E.M., Mandell J.D., Li A., Yang R., Hall B.S., Haberman J.T., Zacharek S.J., Liston C. (2019). Ventral hippocampus interacts with prelimbic cortex during inhibition of threat response via learned safety in both mice and humans. Proc. Natl. Acad. Sci. USA.

[154] Connor D.A., Kutlu M.G., Gould T.J. (2017). Nicotine disrupts safety learning by enhancing fear associated with a safety cue via the dorsal hippocampus. J. Psychopharmacol..

[155] Roozendaal B. (2002). Stress and memory: Opposing effects of glucocorticoids on memory consolidation and memory retrieval. Neurobiol. Learn. Mem..

[156] Vreugdenhil E., Verissimo C.S., Mariman R., Kamphorst J.T., Barbosa J.S., Zweers T., Champagne D.L., Schouten T., Meijer O.C., de Kloet E.R. (2009). MicroRNA 18 and 124a down-regulate the glucocorticoid receptor: Implications for glucocorticoid responsiveness in the brain. Endocrinology.

[157] Hillerer K.M., Slattery D.A., Pletzer B. (2019). Neurobiological mechanisms underlying sex-related differences in stress-related disorders: Effects of neuroactive steroids on the hippocampus. Front. Neuroendocr..

[158] Gurvich C., Thomas N., Kulkarni J. (2020). Sex differences in cognition and aging and the influence of sex hormones. Handb. Clin. Neurol..

[159] Hajali V., Andersen M.L., Negah S.S., Sheibani V. (2019). Sex differences in sleep and sleep loss-induced cognitive deficits: The influence of gonadal hormones. Horm. Behav..

[160] Lambert K.G., Kinsley C.H. (1993). Sex differences and gonadal hormones influence susceptibility to the activity-stress paradigm. Physiol. Behav..

[161] McEwen B.S., Milner T.A. (2017). Understanding the broad influence of sex hormones and sex differences in the brain. J. Neurosci. Res..

[162] Kong F., Zhen Z., Li J., Huang L., Wang X., Song Y., Liu J. (2014). Sex-related neuroanatomical basis of emotion regulation ability. PLoS ONE.

[163] Mahmoud R., Wainwright S.R., Galea L.A. (2016). Sex hormones and adult hippocampal neurogenesis: Regulation, implications, and potential mechanisms. Front. Neuroendocr..

[164] Koss W.A., Frick K.M. (2017). Sex differences in hippocampal function. J. Neurosci. Res..

[165] Scharfman H.E., MacLusky N.J. (2017). Sex differences in hippocampal area CA3 pyramidal cells. J. Neurosci. Res..

[166] Morgan C.P., Bale T.L. (2017). Sex differences in microRNA-mRNA networks: Examination of novel epigenetic programming mechanisms in the sexually dimorphic neonatal hypothalamus. Biol. Sex. Differ..

[167] Murphy S.J., Lusardi T.A., Phillips J.I., Saugstad J.A. (2014). Sex differences in microRNA expression during development in rat cortex. Neurochem. Int..

[168] Sheinerman K., Tsivinsky V., Mathur A., Kessler D., Shaz B., Umansky S. (2018). Age- and sex-dependent changes in levels of circulating brain-enriched microRNAs during normal aging. Aging (Albany N. Y.).

[169] Jirkof P., Bratcher N., Medina L., Strasburg D., Ebert P., Gaskill B.N. (2020). The effect of group size, age and handling frequency on inter-male aggression in CD 1 mice. Sci. Rep..

[170] Svenson K.L., Paigen B. (2019). Recommended housing densities for research mice: Filling the gap in data-driven alternatives. FASEB J..

[171] Nguyen P.V., Abel T., Kandel E.R. (1994). Requirement of a critical period of transcription for induction of a late phase of LTP. Sci. (N. Y.).

[172] Nguyen P.V., Kandel E.R. (1997). Brief theta-burst stimulation induces a transcription-dependent late phase of LTP requiring cAMP in area CA1 of the mouse hippocampus. Learn. Mem. (Cold Spring Harb. N. Y.).

[173] Schneider C.A., Rasband W.S., Eliceiri K.W. (2012). NIH Image to ImageJ: 25 years of image analysis. Nat. Methods.

[174] Prut L., Belzung C. (2003). The open field as a paradigm to measure the effects of drugs on anxiety-like behaviors: A review. Eur. J. Pharmacol..

[175] Ladron de Guevara-Miranda D., Millon C., Rosell-Valle C., Perez-Fernandez M., Missiroli M., Serrano A., Pavon F.J., Rodriguez de Fonseca F., Martinez-Losa M., Alvarez-Dolado M. (2017). Long-lasting memory deficits in mice withdrawn from cocaine are concomitant with neuroadaptations in hippocampal basal activity, GABAergic interneurons and adult neurogenesis. Dis. Model. Mech..

[176] Manas-Padilla M.C., Avila-Gamiz F., Gil-Rodriguez S., Ladron de Guevara-Miranda D., Rodriguez de Fonseca F., Santin L.J., Castilla-Ortega E. (2021). Persistent changes in exploration and hyperactivity coexist with cognitive impairment in mice withdrawn from chronic cocaine. Physiol. Behav..

[177] Bailey K.R., Crawley J.N., Buccafusco J.J. (2009). Anxiety-Related Behaviors in Mice. Methods of Behavior Analysis in Neuroscience.

[178] Manas-Padilla M.C., Gil-Rodriguez S., Sampedro-Piquero P., Avila-Gamiz F., Rodriguez de Fonseca F., Santin L.J., Castilla-Ortega E. (2021). Remote memory of drug experiences coexists with cognitive decline and abnormal adult neurogenesis in an animal model of cocaine-altered cognition. Addict. Biol..

[179] Pinheiro S.H., Zangrossi H., Del-Ben C.M., Graeff F.G. (2007). Elevated mazes as animal models of anxiety: Effects of serotonergic agents. Acad. Bras. Cienc..

[180] Walf A.A., Frye C.A. (2007). The use of the elevated plus maze as an assay of anxiety-related behavior in rodents. Nat. Protoc..

[181] Li H., Zhang C., Shen H., Shen Z., Wu L., Mo F., Li M. (2017). Physiological stress-induced corticosterone increases heme uptake via KLF4-HCP1 signaling pathway in hippocampus neurons. Sci. Rep..

